# Mesenchymal stromal cell-derived exosomes protect against abdominal aortic aneurysm formation through CD74 modulation of macrophage polarization in mice

**DOI:** 10.1186/s13287-024-03808-y

**Published:** 2024-08-04

**Authors:** Jiamin Xu, Jiling Zhao, Haiting Chen, Xi Tan, Wenfeng Zhang, Zhongnan Xia, Dejiang Yao, Yuhua Lei, Biao Xu, Zhonghai Wei, Jiaxin Hu

**Affiliations:** 1 Department of Cardiology, Nanjing Drum Tower Hospital, The Affiliated Hospital of Nanjing University Medical School, Nanjing, China; 2 Cardiovascular Disease Center, The Central Hospital of Enshi Tujia and Miao Autonomous Prefecture, Enshi Clinical College of Wuhan University, No. 158 Wuyang Avenue, Enshi, Hubei China; 3 Hubei Selenium and Human Health Institute, the Central Hospital of Enshi Tujia and Miao Autonomous Prefecture, Enshi, 445000 China; 4 Department of Cardiology, Nanjing Drum Tower Hospital Clinical College of Nanjing University of Chinese Medicine, Nanjing, China; 5 Department of Cardiology, The First Affiliated Hospital of Anhui Medical University, Anhui, China; 6 Surgical Division III, The Central Hospital of Enshi Tujia and Miao Autonomous Prefecture, Enshi Clinical College of Wuhan University, Enshi, Hubei China

**Keywords:** Abdominal aortic aneurysm, Mesenchymal stromal cells, Exosomes, Macrophage polarization, Inflammation

## Abstract

**Background:**

Mesenchymal stromal cell (MSC)-derived exosomes (MSC-Exo) have been recognized for their significant role in regulating macrophage polarization, a process crucial to the pathogenesis of abdominal aortic aneurysm (AAA). However, the therapeutic effects of MSC-Exo on AAA remain largely unexplored. Therefore, this study aimed to investigate the functional and mechanistic aspects of MSC-Exo in the progression of AAA.

**Methods:**

The MSC-derived exosomes were characterized using Transmission Electron Microscopy, Nanoparticle Tracking Analysis, and Western blotting. An experimental mouse model of AAA was established through the administration of angiotensin II (Ang II) in male apoe^−/−^ mice and calcium chloride (CaCl_2_) in male C57/B6 mice, with subsequent tail vein injection of exosomes to evaluate their efficacy against AAA. Macrophage polarization was assessed using immunofluorescence staining and WB analysis. Mechanistic analysis was performed using 4D Label-free Proteomics analysis.

**Results:**

We found that intravenous administration of MSC-Exo induced M2 polarization of macrophages within an inflammatory environment, effectively impeding AAA development in Ang II or CaCl_2_-induced AAA model. The therapeutic efficacy of MSC-Exo treatment was dependent on the presence of macrophages. Mechanistically, MSC-Exo suppressed the levels of cluster of differentiation 74 (CD74), modulating macrophage polarization through the TSC2-mTOR-AKT pathway. These findings highlight the potential of MSC-Exo as a therapeutic strategy for AAA by modulating macrophage polarization.

**Supplementary Information:**

The online version contains supplementary material available at 10.1186/s13287-024-03808-y.

## Introduction

Abdominal aortic aneurysm (AAA) is characterized by the localized dilation of the abdominal aorta, reaching or exceeding 50% of the normal arterial diameter [1]. This condition poses a severe threat to life, as rupture of the aorta can lead to mortality rates as high as 90% [2]. Despite notable progress in surgical interventions for AAA, effective therapeutic agents remain limited, underscoring the imperative to enhance our comprehension of the underlying disease mechanisms.

Inflammation assumes a pivotal role in the advancement of AAA, which is increasingly acknowledged as a fundamentally chronic inflammatory disorder [3]. This inflammatory response arises from the accumulation and activation of various inflammatory cell populations, including macrophages [4]. During the initial stages of AAA development, M1 macrophages contribute to disease progression by generating pro-inflammatory cytokines and matrix metalloproteinases, thereby fostering AAA formation. Subsequently, M2 macrophages demonstrate anti-inflammatory properties and contribute to matrix remodeling and tissue repair [5]. Notably, investigations have revealed that Circular RNA Cdyl facilitates the development of AAA by promoting M1 macrophage polarization and instigating an M1-type inflammatory response [6]. Moreover, the transition from pro-inflammatory M1 macrophages to anti-inflammatory M2 macrophages has been implicated in modulating vascular inflammation within AAA [7]. Accordingly, early intervention targeting macrophage polarization holds promise for impeding excessive AAA progression or rupture, rendering it a potential therapeutic target for AAA treatment.

Mesenchymal stromal cells (MSCs) possess multipotent characteristics and exhibit regenerative and immunomodulatory functions [8]. Notably, in recent years, MSC-based therapy has demonstrated promising efficacy in various cardiovascular ailments. Numerous studies have substantiated the inhibitory effects of MSCs on AAA formation through their modulation of the inflammatory response and mitigation of aortic elastin degradation [9, 10]. Moreover, emerging evidence underscores the capacity of MSC transplantation to promote M2 macrophage polarization [11]. The therapeutic benefits attributed to MSCs primarily arise from their paracrine effects, wherein they release various factors that contribute to tissue repair and immune regulation [12]. MSC-derived exosomes (MSC-Exo), lipid bilayer extracellular vesicles secreted by MSCs, play a critical role in promoting the therapeutic effects of MSCs [13]. Preclinical research suggests that MSC-Exo exhibit anti-inflammatory and immunomodulatory properties [14, 15]. However, it is unknown whether MSC-Exo could protect against AAA by inducing macrophages polization towards an anti-inflammatory phenotype. Previous research has indicated that the TSC2-mTOR-AKT pathway, a crucial metabolic signaling pathway, plays a significant role in modulating macrophage polarization [16]. In this study, we utilized an AngII and CaCl_2_-induced AAA model and found that MSC-Exo effectively attenuated AAA formation by regulating macrophage polarization. This was achieved through down-regulation of CD74, leading to the inhibition of PKM2 expression and modulation of the TSC2-mTOR-AKT pathway.

## Methods

### Animal experimental protocol

Apoe^−/−^ and C57BL/6 mice (8–10 weeks old) were procured from the Model Animal Research Centre of Nanjing University. The mice were housed under a 12-hour dark/light cycle with controlled environmental conditions of temperature (22 ± 1℃) and humidity (65–70%). They were provided with standard laboratory diet and water ad libitum. The mice were anesthetized using isoflurane (1.5-2%) and cervical dislocation was employed for euthanizing the mice. The work has been reported in line with the ARRIVE guidelines 2.0.

### Cell experimental protocol

Peritoneal macrophages were exposed to lipopolysaccharide (LPS, Sigma-Aldrich) at a concentration of 100 ng/ml for 6 h to induce the M1 phenotype. Following this, the culture medium was replaced with fresh medium. The cells were then incubated with either MSC-Exo at a concentration of 40 µg/ml or phosphate-buffered saline (PBS) for an additional 24 h. Untreated cells served as the negative control. After 24 h, the supernatants were collected for ELISA assay, while the cells were harvested for immunofluorescence staining as well as RNA and protein extraction.

### Exosome isolation and characterization

When mesenchymal stem cells (MSCs) reached 70–80% confluency, the culture medium was replaced with medium containing 5% exosome-depleted fetal bovine serum (FBS). The cells were then cultured for 48 h. Exosomes were isolated using a differential centrifugation method: the cell culture supernatants were first centrifuged at 3,000 g for 25 min and then at 10,000 g for 1 h at 4 °C to remove cellular debris and shedding vesicles. Subsequently, the supernatant was centrifuged at 100,000 g for 3 h at 4 °C, and the resulting pellet was resuspended in phosphate-buffered saline (PBS).

The morphology of exosomes was visualized using a transmission electron microscope (JEM-1011, Japan). The concentration and size of the exosomes were assessed using the NanoSight NS300 system (Malvern, UK). Protein markers including CD63, CD9, and TSG101 were evaluated by western blot to confirm the presence of exosomes. The total protein concentration of the exosomes was quantified using the BCA assay (Thermo Scientific).

### Statistical analysis

GraphPad Prism (version 9.5) software was employed for all statistical analyses. Quantitative data are presented as mean ± standard deviation (SD). Comparisons between multiple groups were performed using one-way ANOVA, while comparisons between two groups were conducted using Student’s t-test. The D’Agostino-Pearson omnibus normality test or the Shapiro-Wilk normality test was utilized to assess whether the data followed a normal distribution. Statistical significance was considered at a P value < 0.05, and all tests were two-tailed. The significance levels were denoted as follows: *P < 0.05, **P < 0.01, ***P < 0.001, ****P < 0.0001; ns represented non-significant results.

Specific experimental methods are detailed in the supplementary materials section of our study. Please refer to the supplementary materials for a comprehensive description of the experimental procedures.

## Results

### The characterization of MSC-Exo

**Fig. 1:**
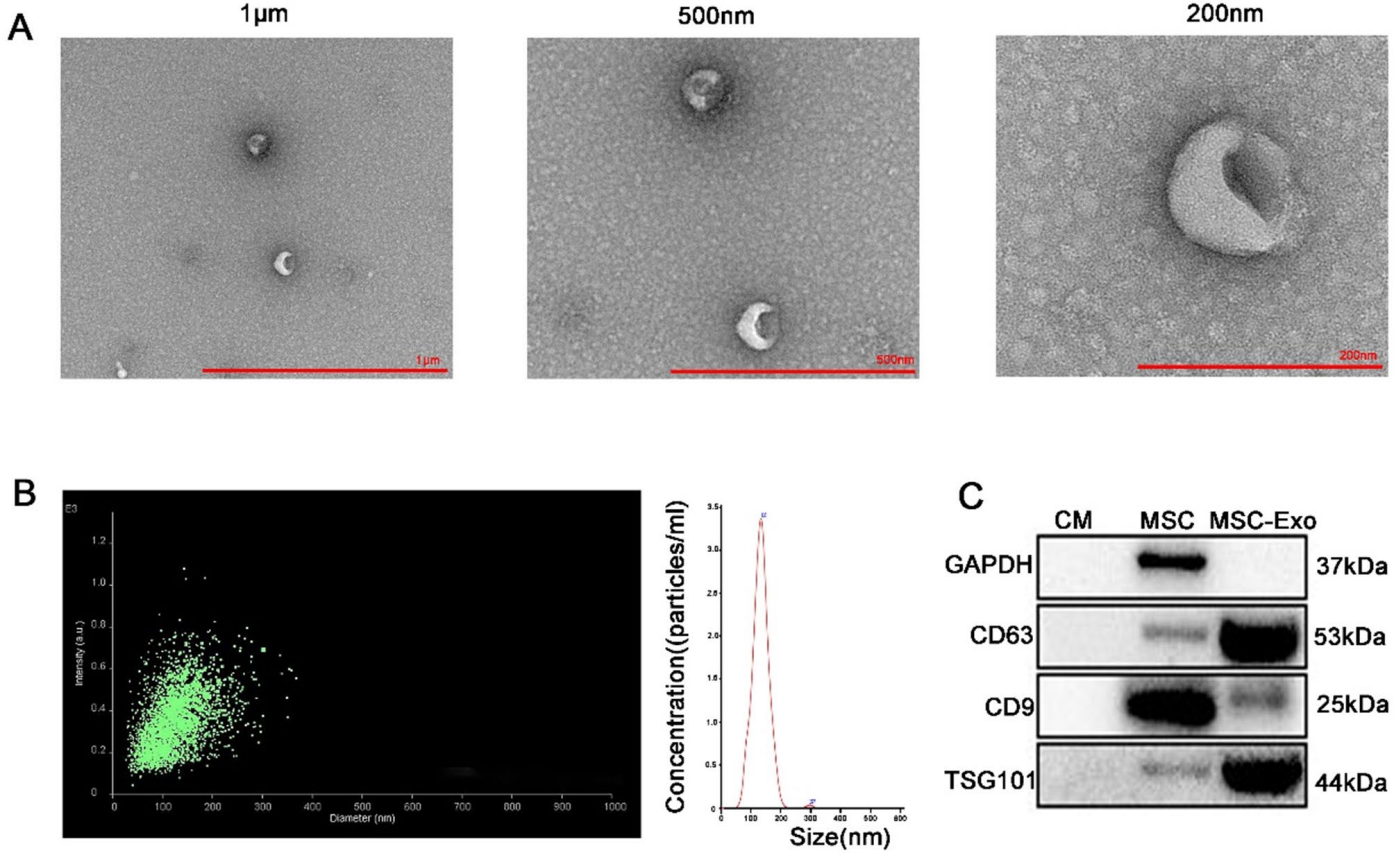
The MSC-derived exosomes were characterized in this study. (**A**) Transmission electron microscopy was performed to visualize the morphology of MSC-derived exosomes, showing images at different scales (1 μm, 500 nm, and 200 nm). (**B**) Nanoparticle tracking analysis was used to determine the size distribution of MSC-derived exosomes. (**C**) Western blot analysis was conducted to confirm the presence of exosomal markers, including CD63, CD9, and TSG101, in the conditioned media (CM), MSC lysates, and MSC-derived exosomes. Full-length blots/gels are presented in Supplementary Fig. 1

Exosomes were purified from MSC culture supernatants by gradient centrifugation and characterised by transmission electron microscopy, nanoparticle tracking analysis (NTA) and western blot analysis. First, transmission electron microscopy showed that the isolated vesicles had a bilayer membrane structure with a typical cup-shaped morphology (Fig. 1A). NTA showed that the size distribution of the vesicles ranged from 50 to 150 nm (Fig. 1B). Western blot analysis of the vesicles further confirmed the expression of exosomal markers such as CD63, CD9, and TSG101 (Fig. 1C). These data indicated that what we had isolated from MSC supernatants were indeed exosomes.

### MSC-Exo attenuates angiotensin II (AngII)-induced AAA formation and promotes M2 macrophage polarization in Apoe^−/−^ mice

**Fig. 2:**
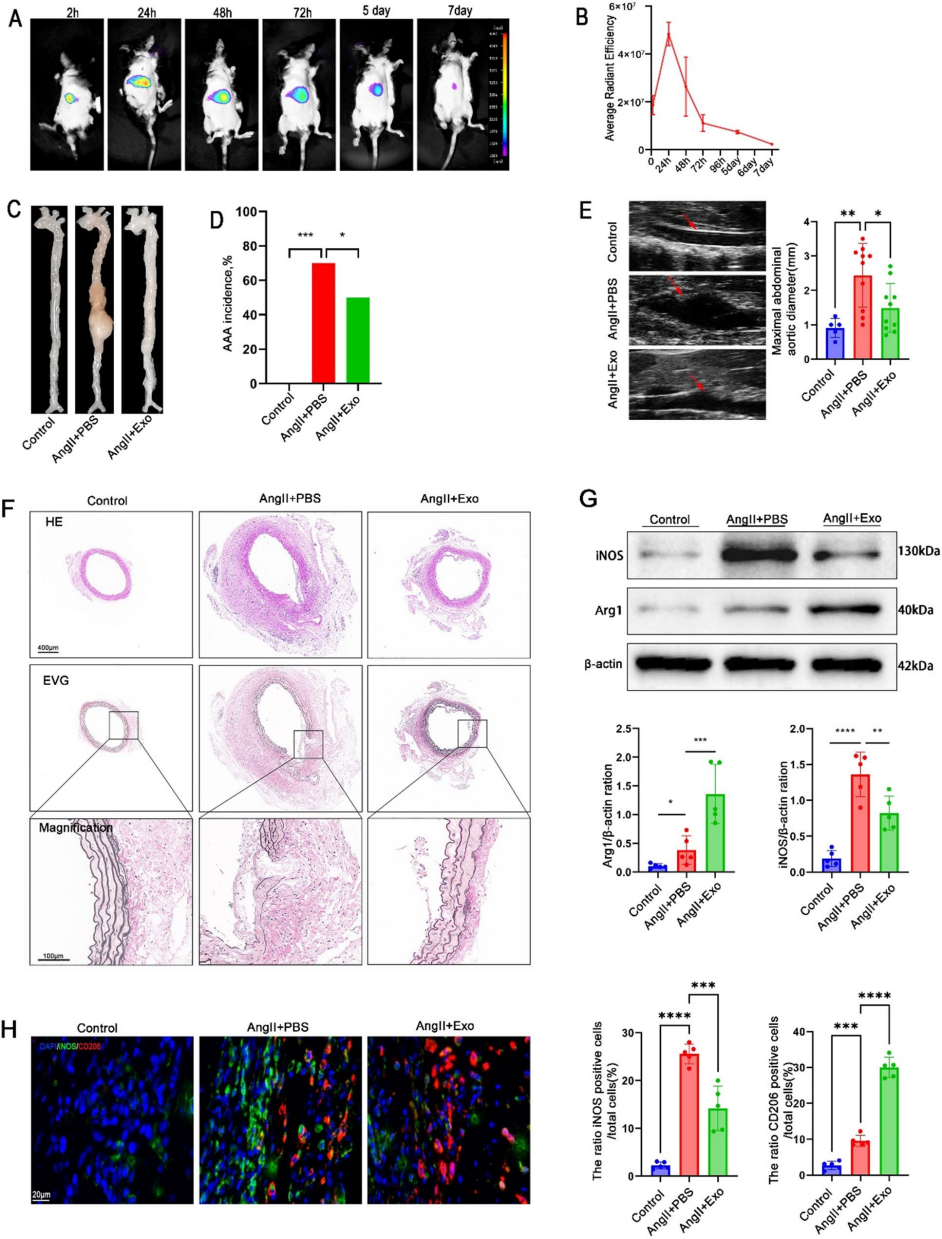
The effects of intravenous administration of MSC-Exo on AngII-induced AAA formation and macrophage polarization in mice. (**A**) In vivo imaging was performed after injecting DiR-labeled MSC-Exo into the mouse tail veins, showing fluorescence distribution. (**B**) The average radiant efficiency was calculated to quantify the signals from the labeled exosomes. (**C**) Macroscopic images of the aorta were captured in AngII-induced AAA mice treated with MSC-Exo or PBS. (**D**) The incidence of AAA was determined in AngII-induced mice treated with MSC-Exo or PBS. (**E**) Abdominal ultrasound imaging was conducted to measure the maximal abdominal aortic diameter in PBS- and MSC-Exo-treated AngII-induced AAA mice. (**F**) Histological analysis was performed using H&E and EVG staining of the abdominal aortas in PBS- and MSC-Exo-treated AngII-induced AAA mice (magnified photographs included). (**G**) Western blot analysis was conducted to assess the expression levels of iNOS and Arg1 in the abdominal aortas of PBS- and MSC-Exo-treated AngII-induced AAA mice, Full-length blots/gels are presented in Supplementary Fig. 2. (**H**) Immunofluorescence staining was performed in the abdominal aortas of PBS- and MSC-Exo-treated AngII-induced AAA mice, using CD206 (red), iNOS (green), and DAPI. (blue) (scale bars, 20 μm). *P < 0.05; **P < 0.01; ***P < 0.001; ****P < 0.0001

To evaluate the impact of MSC-Exo on angiotensin II (AngII)-induced AAA formation, we investigated the duration of MSC-Exo presence in mice. In vivo imaging using DiR-labeled MSC-Exo revealed effective uptake and sustained presence in the mouse for up to 7 days (Fig. 2A, B). Thus, intravenous administration of MSC-Exo or phosphate-buffered saline (PBS) was performed every 7 days, starting from the day of AngII injection. Aortic tissue samples were collected on day 28 after AngII injection. Due to budget constraints, we used a total of 25 mice for the experiment. This sample size is sufficient to support our conclusions.25 male apoe^−/−^ mice were randomly assigned into three groups: control group, AngII + PBS group, and AngII + Exo group. The AngII + Exo group received intravenous injection of MSC-Exo (*n* = 10) at a dose of 3 µg/g, while the AngII + PBS group received the same dose of PBS (*n* = 10), both concurrent with AngII infusion (1000 ng/kg/min) for 4 weeks. An additional 5 male apoe^−/−^ mice were injected with PBS and infused with saline for 4 weeks as controls. No AAA formation was observed in the control group following tail vein injection with PBS (Fig. 2C, D). In the AngII + Exo group, the incidence of AAA was 50% (5/10), significantly lower than the AngII + PBS group (70%, 7/10) (Fig. 2D). Abdominal ultrasound examination further confirmed that the maximal diameter of the abdominal aorta in the AngII + PBS group was significantly higher compared to the control group(2.44 verus 0.91 ± 0.4285 cm, 95%CI:0.6043 to 2.456), while intervention with MSC-Exo noticeably decreased the maximal diameter of the abdominal aorta in AngII-induced mice (2.44 verus 1.49 ± 0.3678 cm,95%CI:-1.726 to -0.1737) (Fig. 2E). Moreover, histological analysis revealed reduced degradation of elastic fibers in the vessel wall of AngII + Exo mice compared to AngII + PBS mice (Fig. 2F). These findings demonstrate that MSC-Exo mitigated the development of AngII-induced AAA.

In order to investigate the potential effects of MSC-derived exosomes (MSC-Exo) on macrophage polarization in AngII-induced AAA mice, we examined whether MSC-Exo could influence the phenotypes of macrophages towards M1 or M2 polarization. Western blot analysis demonstrated a significant decrease in the M1 marker iNOS expression and an increase in the M2 marker Arg1 expression in the AngII + Exo group compared to the AngII + PBS group (Fig. 2G). Additionally, immunofluorescence staining further confirmed a significant reduction in the M1 marker iNOS and an elevation in the M2 marker CD206 following MSC-Exo treatment (Fig. 2H). Collectively, these findings suggest that in AngII-induced AAA mice, MSC-Exo promotes the polarization of macrophages towards the M2 phenotype.

### MSC-Exo alleviates calcium chloride (CaCl_2_)-induced AAA formation and promotes M2 macrophage polarization in C57/B6 mice

**Fig. 3:**
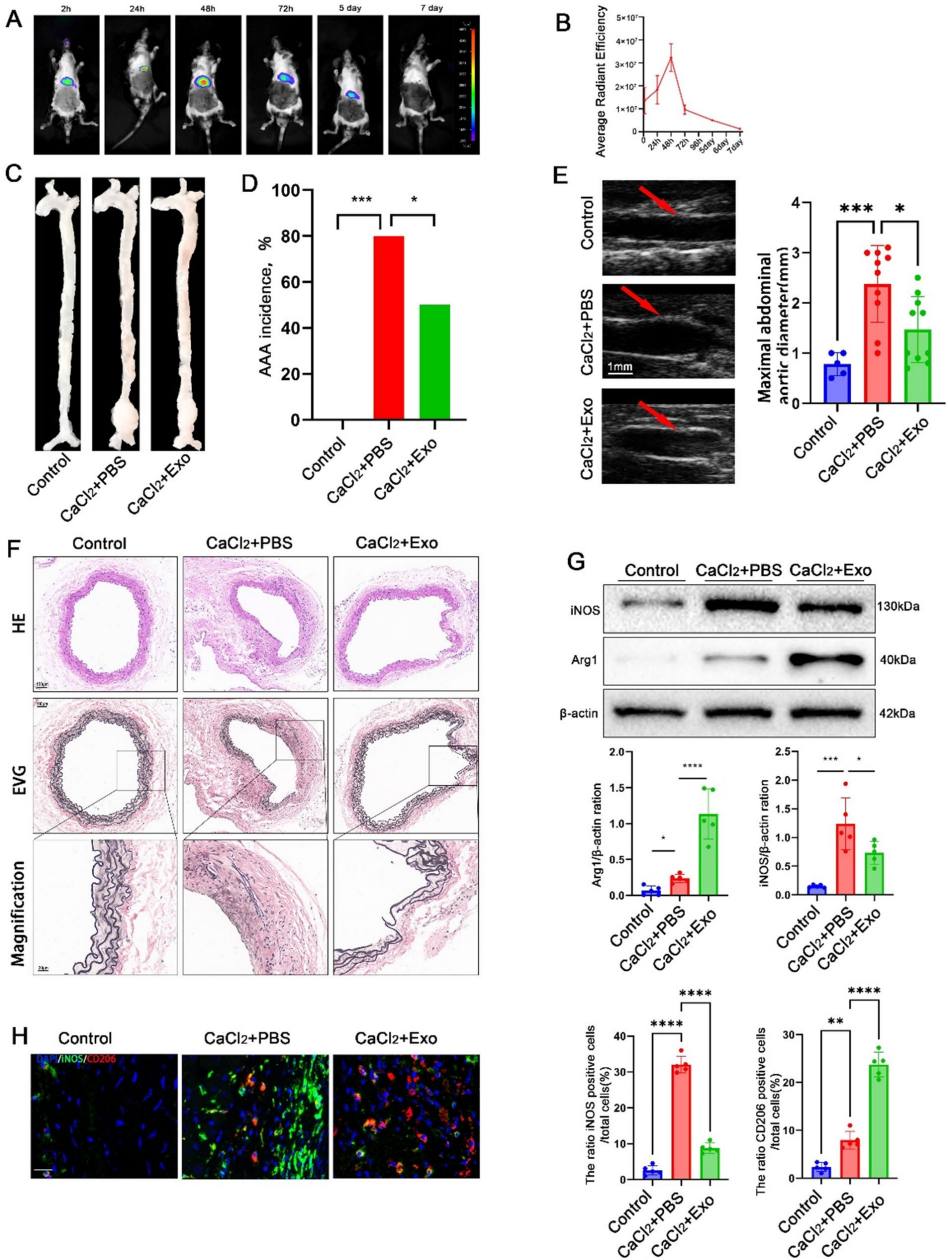
The effects of intravenous administration of MSC-Exo on CaCl_2_-induced AAA formation and macrophage polarization in mice. (**A**) Mouse in vivo imaging was performed after injecting DiR-labeled MSC-Exo, displaying the fluorescence distribution. (**B**) The average radiant efficiency was quantified to assess the signals from the labeled exosomes. (**C**) Macroscopic images of the aorta were captured in CaCl_2_-induced AAA mice treated with MSC-Exo or PBS. (**D**) The incidence of AAA was determined in CaCl_2_-induced mice treated with MSC-Exo or PBS. (**E**) Abdominal ultrasound imaging was conducted to measure the maximal abdominal aortic diameter in PBS- and MSC-Exo-treated CaCl_2_-induced AAA mice. (**F**) Histological analysis was performed using H&E and EVG staining of the abdominal aortas in PBS- and MSC-Exo-treated CaCl_2_-induced AAA mice (including magnified photographs). (**G**) Western blot analysis was conducted to evaluate the expression levels of iNOS and Arg1 in the abdominal aortas of PBS- and MSC-Exo-treated CaCl_2_-induced AAA mice, full-length blots/gels are presented in Supplementary Fig. 3. (**H**) Immunofluorescence staining was performed in the abdominal aortas of PBS- and MSC-Exo-treated CaCl_2_-induced AAA mice, employing CD206 (red), iNOS (green), and DAPI. (blue) (scale bars, 20 μm). *P < 0.05; **P < 0.01; ***P < 0.001; ****P < 0.0001

To further investigate the impact of MSC-derived exosomes (MSC-Exo) on AAA formation, we utilized the CaCl_2_-induced AAA model. 25 male C57/B6 mice were randomly assigned into three groups: control group, CaCl_2_ + PBS group, and CaCl_2_ + Exo group.The CaCl_2_ + Exo group mice were intravenously injected with MSC-Exo (*n* = 10) at a dose of 3 µg/g and the CaCl_2_ + PBS group injected the same amount of PBS (*n* = 10) and subsequently administered CaCl_2_. Four weeks later, the mice were sacrificed for analysis. An additional 5 male WT mice received intravenous injection of PBS and saline infusion as controls. In vivo imaging revealed the uptake of DiR-labeled MSC-Exo by the CaCl_2_-induced AAA mice (Fig. 3A, B). No evidence of AAA formation or significant morphological changes in the abdominal aorta were observed in the control group (Fig. 3C, D, F). The incidence of AAA in the CaCl_2_ + Exo group was 50% (5/10), significantly lower than that in the CaCl_2_ + PBS group (80%, 8/10) (Fig. 3D). Additionally, the maximal diameter of the abdominal aorta in CaCl_2_ + Exo mice was significantly smaller compared to CaCl_2_ + PBS mice (1.47verus 2.38 ± 0.319 cm,95%CI: 0.2399 to 1.580)(Fig. 3E). Consistent with the AngII-induced AAA model, CaCl_2_ + Exo mice exhibited reduced elastic fiber degradation in the vessel wall compared to CaCl_2_ + PBS mice (Fig. 3F). These findings demonstrate that MSC-Exo intervention alleviated AAA formation in the CaCl_2_-induced AAA model when compared to CaCl_2_ + PBS mice.

Subsequently, we examined the effects of MSC-Exo on macrophage polarization in CaCl_2_-induced AAA mice. Through western blot analysis (Fig. 3G) and immunofluorescence staining (Fig. 3H) of M1 and M2 protein expression, it was observed that the M1 marker expression was significantly reduced, whereas the M2 marker expression was increased following MSC-Exo intervention. These findings collectively indicate that MSC-Exo promotes M2 macrophage polarization in CaCl_2_-induced AAA mice.

### Systemic depletion of macrophages reduces the efficacy of MSC-Exo treatment

**Fig. 4:**
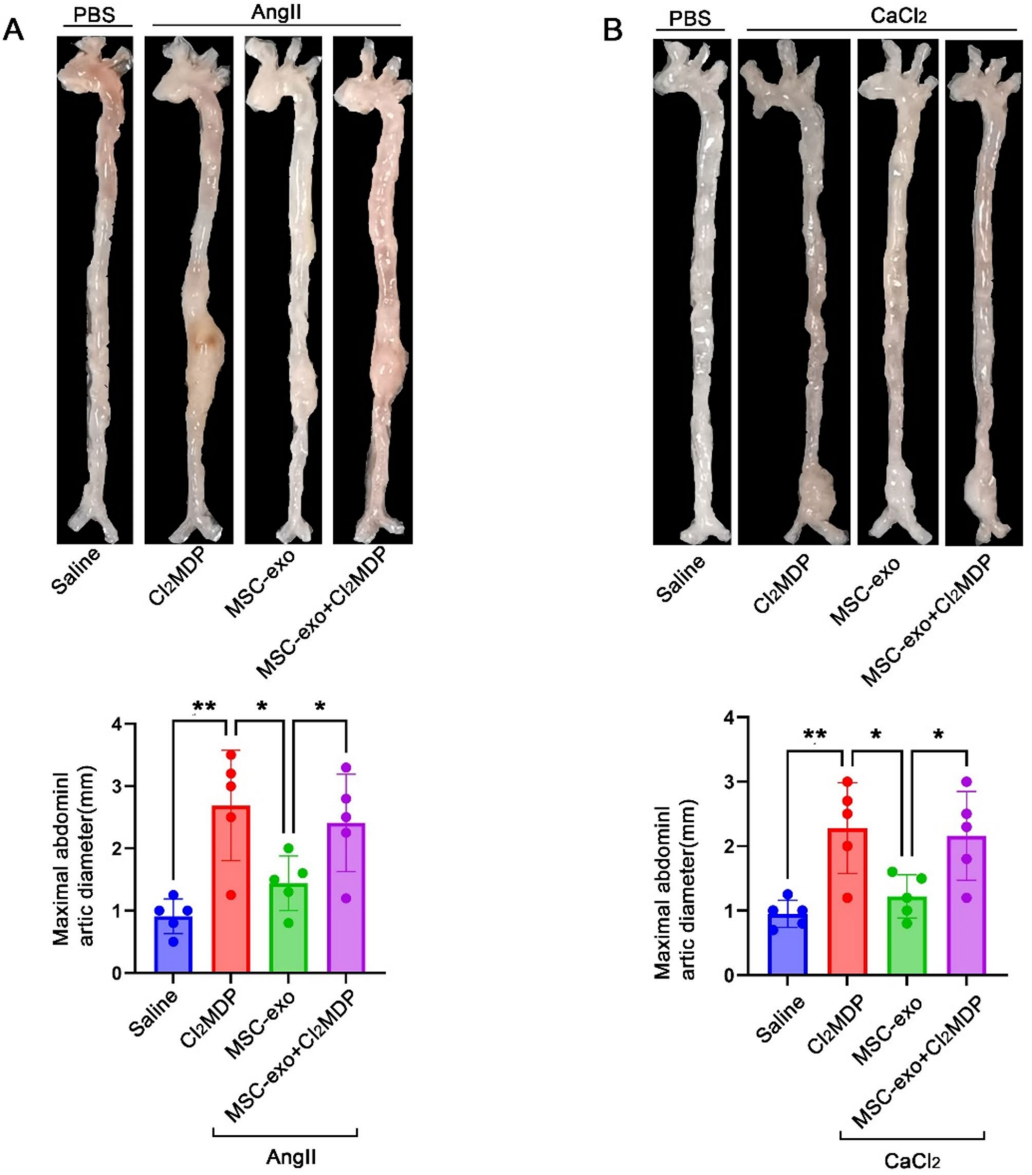
Systemic depletion of macrophages reduces the efficacy of MSC-Exo treatment. (**A**) Macroscopic images of the aorta and maximal abdominal aortic diamete were assessed in AngII-induced AAA mice. (**B**) Similarly, macroscopic images of the aorta and maximal abdominal aortic diamete in CaCl_2_-induced AAA mice (per group *n* = 5) ***P < 0.001; ****P < 0.0001; ns, not significant

To explore the role of macrophages in the efficacy of MSC-derived exosomes (MSC-Exo), we depleted macrophages using clodronate liposomes. Flow cytometry analysis confirmed a significant reduction in macrophage populations in the spleen, blood, and aorta upon clodronate liposome treatment (Supplementary material online, Figure S1). In AngII-induced AAA mice, the diameter of AAAs showed a significant increase in the group of mice treated with exosomes and clodronate liposomes compared to the group treated with exosomes alone(2.41verus 1.44 ± 0.4009 cm,95%CI: 0.04558 to 1.894).(Fig. 4A) .Similar results were observed in the CaCl_2_-induced AAA model(2.16verus 1.22 ± 0.3421 cm,95%CI: 0.1512 to 1.729) (Fig. 4B). These findings indicate that the depletion of macrophages impairs the protective efficacy of MSC-Exo, suggesting that macrophages play a crucial role in mediating the beneficial effects of MSC-Exo against AAA formation.

### MSC-Exo promoted macrophage conversion to M2 phenotype in inflammatory environment in vitro

**Fig. 5:**
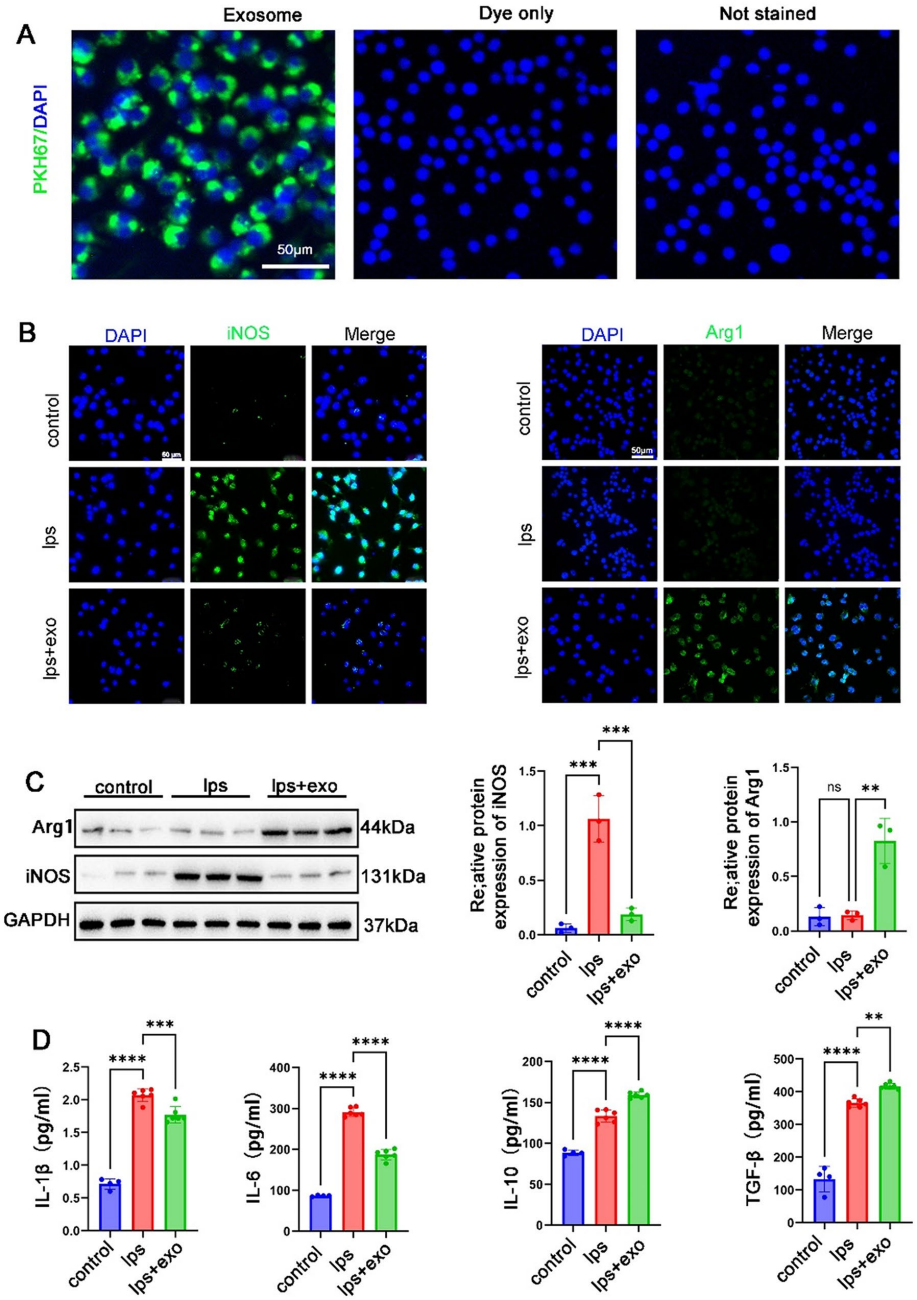
MSC-Exo promoted the conversion of macrophages to M2 phenotype in inflammatory environment in vitro. (**A**) Fluorescence imaging showing the uptake of PKH67-labeled exosomes (green) by macrophages, with DAPI staining for nuclei (blue). The “Dye only” control represents macrophages stained with PKH67 alone, while “Not stained” indicates macrophages without fluorescence labeling. Scale bar = 50 μm. (**B**) Immunofluorescence images depicting the expression of iNOS and Arg1 in LPS-stimulated macrophages after 24 h of culture with MSC-Exo or PBS. *n* = 3, Scale bar = 50 μm. (**C**) Western blot analysis and statistical analysis of iNOS and Arg1 protein levels in LPS-stimulated cells cultured with MSC-Exo or PBS. (*n* = 3, Full-length blots/gels are presented in Supplementary Fig. 5). (**D**) Concentration of cytokines associated with M1 markers (IL-1β and IL-6) and M2 markers (IL-10 and TGF-β) in the supernatants of LPS-stimulated cells cultured with MSC-Exo or PBS. *n* = 5. **P < 0.01; ***P < 0.001; ****P < 0.0001; ns, not significant

In order to further investigate the impact of MSC-derived exosomes (MSC-Exo) on macrophage polarization, we treated peritoneal macrophages with MSC-Exo. To assess the internalization of MSC-Exo by macrophages, PKH67-labeled MSC-Exo was incubated with peritoneal macrophages for 6 h. Immunofluorescence images demonstrated efficient uptake of PKH67-labeled MSC-Exo by peritoneal macrophages (Fig. 5A). Lipopolysaccharide (LPS) was then added to the culture medium to induce an inflammatory environment, followed by the addition of MSC-Exo to the LPS-stimulated macrophages. After 24 h, the concentrations of pro-inflammatory and anti-inflammatory cytokines in the supernatants, as well as the protein levels of iNOS and Arg1, were assessed. Immunofluorescence staining revealed that MSC-Exo inhibited LPS-induced iNOS production and enhanced the upregulation of Arg1 (Fig. 5B). Western blot analysis demonstrated consistent changes with the immunofluorescence staining (Fig. 5C). Furthermore, the levels of pro-inflammatory cytokines IL-1β and IL-6, as well as anti-inflammatory cytokines IL-10 and TGF-β, were measured in the supernatants (Fig. 5D). The levels of these inflammatory cytokines were elevated in the supernatants of the LPS group compared to the control group. However, MSC-Exo attenuated the LPS-induced increase in pro-inflammatory cytokines (IL-1β and IL-6) and promoted the upregulation of anti-inflammatory cytokines (IL-10 and TGF-β). Collectively, these results indicate that MSC-Exo facilitated the conversion of macrophages to the M2 phenotype.

### CD74 was a candidate effector of MSC-Exo mediated macrophage polarization

**Fig. 6:**
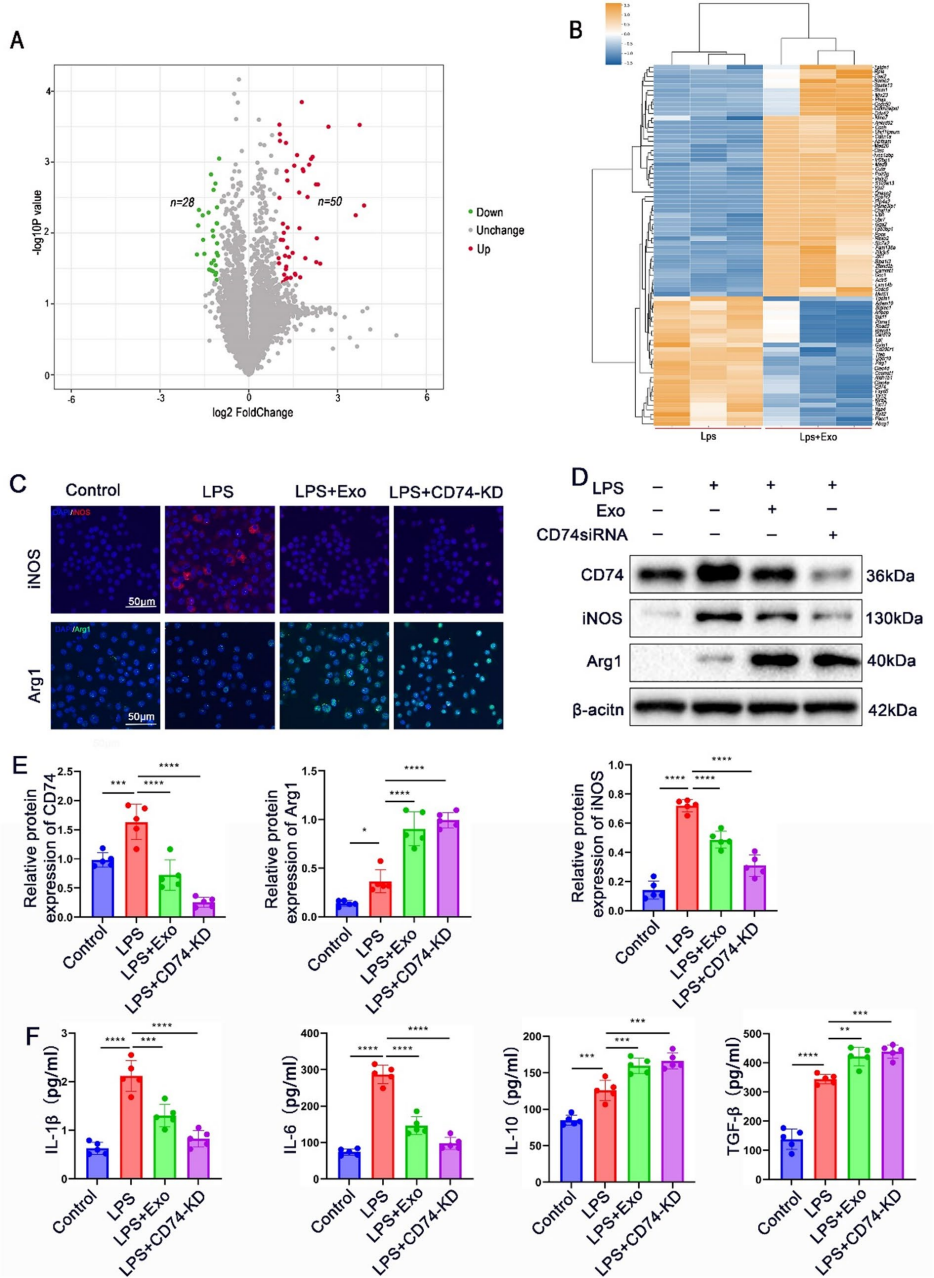
CD74 is involved in MSC-Exo mediated macrophage polarization in vitro. (**A**) Volcano plot comparing the differentially expressed proteins between the LPS+Exo group and the LPS group, with red indicating up-regulated proteins and green indicating down-regulated proteins. (**B**) Heat map displaying the differential expression of the top 50 up-regulated and top 28 down-regulated proteins between the LPS+Exo group and the LPS group. （**C**）Representative immunofluorescence images illustrating the expression of iNOS and Arg1 in LPS-stimulated peritoneal macrophages cultured with MSC-Exo or CD74 siRNA. iNOS is shown in red, Arg1 in green, and DAPI staining for nuclei in blue. Scale bars = 50 μm. (**D**-**E**) Western blot analysis (**D**) and statistical analysis (**E**) of CD74, iNOS, and Arg1 protein levels in LPS-stimulated cells cultured with MSC-Exo or CD74 siRNA(Full-length blots/gels are presented in Supplementary Figure 6). (**F**) Concentration of cytokines associated with M1 markers (IL-1β and IL-6) and M2 markers (IL-10 and TGF-β) in the supernatants of LPS-stimulated macrophages cultured with MSC-Exo or CD74 siRNA. (per group n = 5). *P < 0.05; **P < 0.01; ***P < 0.001; ****P < 0.0001

To elucidate the mechanisms underlying the regulation of macrophage polarization by MSC-derived exosomes (MSC-Exo), LPS-stimulated peritoneal macrophages were treated with PBS or MSC-Exo. Label-free proteomics analysis was conducted to identify differentially expressed proteins. The volcano plot (Fig. 6C) and heat map (Fig. 6B) demonstrated 78 differentially expressed proteins in the LPS + Exo group compared to the LPS + PBS group, consisting of 50 up-regulated proteins and 28 down-regulated proteins. Notably, the top 6 most significantly up-regulated proteins were Jpt1, Ppp1r2, S100a13, Psme3ip1, Ccnh, and Dnase2 (Figure S2A). Conversely, the top 6 most significantly down-regulated proteins were Rsad2, Tgln1, Clec4e, Fxyd5, Klra2, and CD74 (Figure S3A). Subsequently, siRNAs targeting the 6 most significantly up-regulated proteins were applied to LPS-stimulated peritoneal macrophages cultured with MSC-Exo. However, their knockdown did not affect the effect of MSC-Exo on macrophage polarization, suggesting that these proteins are not direct targets of MSC-Exo for macrophage polarization (Figure S2B). On the other hand, the effects of the 6 most significantly down-regulated proteins on macrophage polarization were investigated. qRT-PCR results confirmed that knockdown of CD74, among these proteins, exhibited a similar effect as MSC-Exo, promoting the conversion of macrophages to the M2 phenotype under an inflammatory environment, while the other 5 down-regulated proteins did not show the same effect (Figure S3B). Additional, tissue immunofluorescence staining revealed a notable increase in CD74 expression in the abdominal aorta tissues of mice treated with AngII and CaCl2, compared to the control group. However, following exosome treatment, there was a significant decrease in CD74 expression(Figure S4). These data indicated that CD74 may be a candidate effector of MSC-Exo mediated macrophage polarization.

To validate the previous findings at the protein level, immunofluorescence staining was performed and demonstrated that the M1 marker iNOS was significantly reduced while the M2 marker Arg1 was elevated after MSC-Exo treatment. Similarly, CD74 siRNA recapitulated the effect of MSC-Exo on macrophage polarization (Fig. 6C). Western blot analysis further confirmed that CD74 siRNA effectively reduced CD74 protein expression and replicated the effect of MSC-Exo on promoting macrophage polarization towards the M2 phenotype (Fig. 6D-E). Additionally, the levels of pro-inflammatory cytokines IL-1β and IL-6, as well as anti-inflammatory cytokines IL-10 and TGF-β, were measured in the supernatants (Fig. 6F). The LPS group exhibited increased levels of these inflammatory cytokines compared to the control group. However, both MSC-Exo and CD74 siRNA inhibited the LPS-induced increase in pro-inflammatory cytokines (IL-1β and IL-6) and enhanced the upregulation of anti-inflammatory cytokines (IL-10 and TGF-β). These results collectively suggest that CD74 is a potential effector involved in MSC-Exo-mediated macrophage polarization.

### Inhibition of CD74 activated the TSC2-mTOR -AKT pathway

**Fig. 7:**
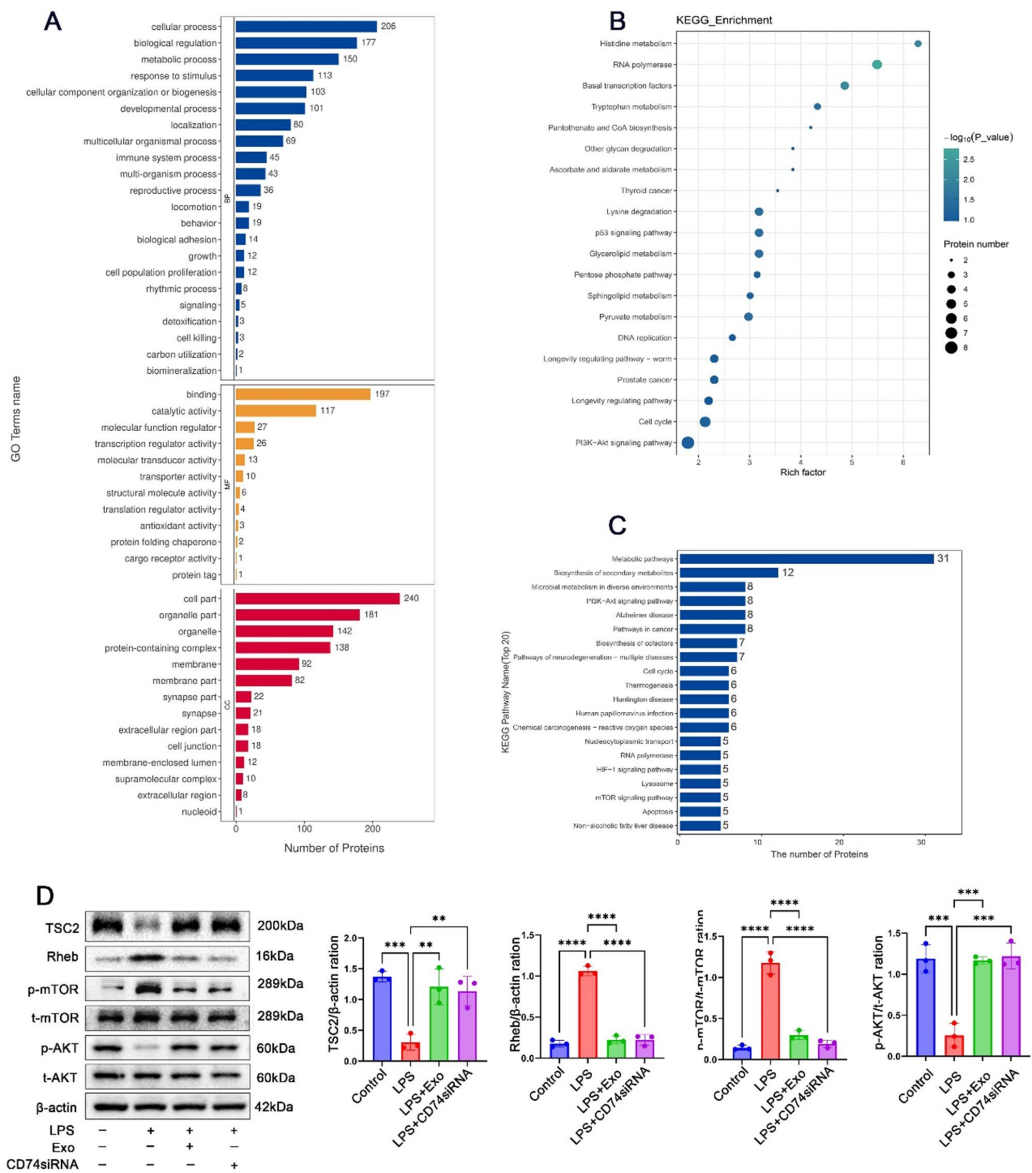
CD74 was involved in the TSC2-mTOR-AKT signalling cascades in peritoneal macrophages. (**A**) The names of significantly enriched Gene Ontology terms from the Gene Ontology analysis.(**B**-**C**) KEGG pathway enrichment analysis results showing the top 20 pathways with their enrichment scores. The rich factor represents the degree of enrichment of the differentially expressed proteins, and the p-value is indicated by a color scale. (**D**) Representative images of western blots and statistical analysis demonstrating the TSC2-Mtor-AKT signaling pathway in LPS-stimulated peritoneal macrophages treated with MSC-Exo or CD74 siRNA. (per group *n* = 3, Full-length blots/gels are presented in Supplementary Fig. 7) *P < 0.05; **P < 0.01; ***P < 0.001; ****P < 0.0001

To gain further insights into the regulatory mechanisms of CD74 on macrophage phenotype, bioinformatics analysis was conducted. Gene ontology analysis (Fig. 7A) and KEGG pathway enrichment analysis (Fig. 7B-C) revealed that the differentially expressed proteins were mainly involved in metabolic pathways. Notably, the TSC2-mTOR-AKT signaling pathway has been implicated in the regulation of macrophage polarization^17^. Therefore, we investigated the effects of MSC-Exo and CD74 siRNA on TSC2 and its downstream signaling pathways using peritoneal macrophages (Fig. 7D). In vitro experiments demonstrated that MSC-Exo treatment in an inflammatory environment resulted in significant upregulation of TSC2 and Akt levels, while Rheb and mTOR expression levels were downregulated. Similarly, treatment with CD74 siRNA activated the TSC2-mTOR-AKT pathway. These findings suggest that CD74 is involved in the modulation of the TSC2-mTOR -AKT signaling cascade.

### CD74 regulated macrophage polarization by interacting with PKM2

**Fig. 8:**
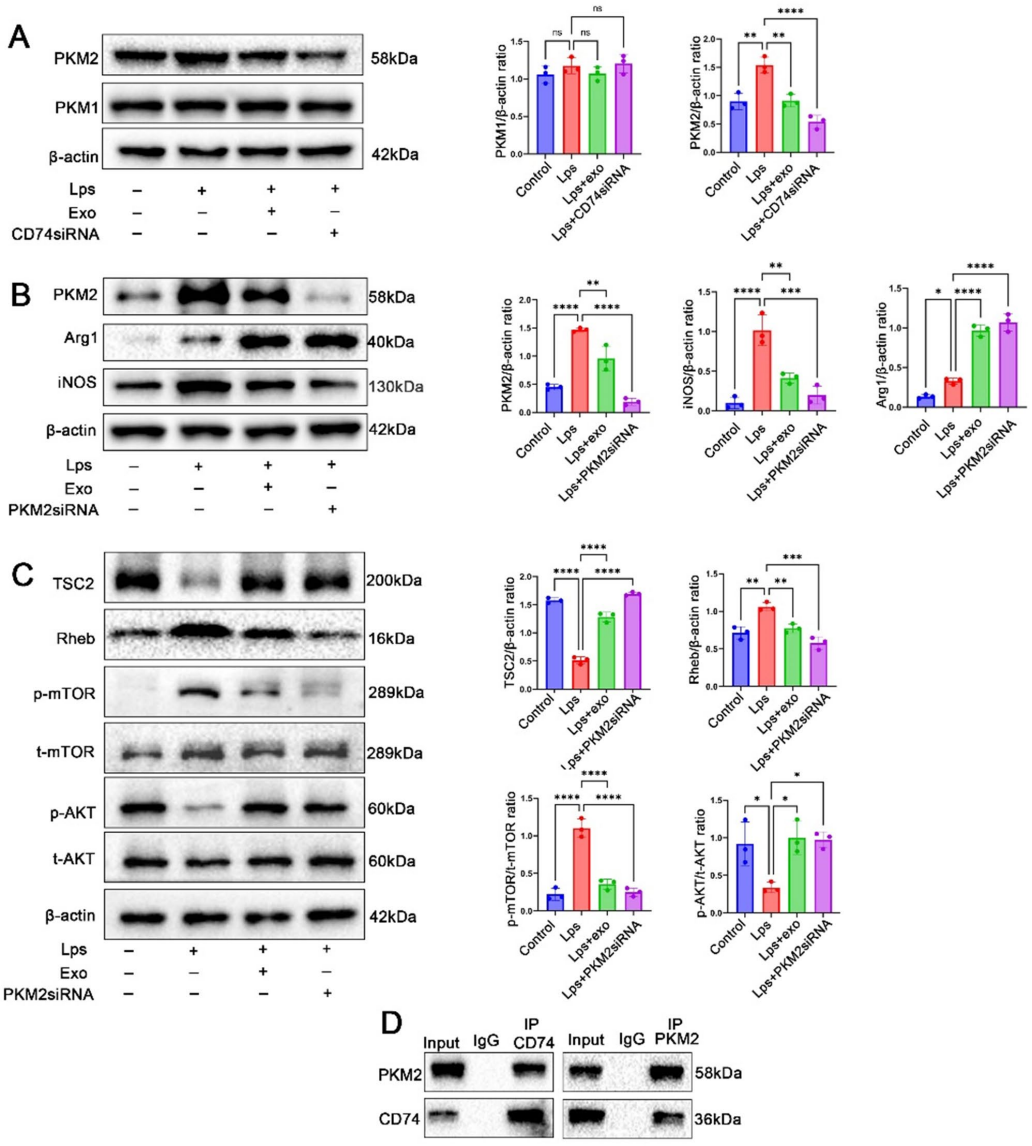
CD74 modulates peritoneal macrophages phenotype through targeting PKM2. (**A**) Representative images of western blots and statistical analysis for protein levels of PKM1 and PKM2 in LPS-stimulated peritoneal macrophages treated with MSC-Exo or CD74 siRNA. (**B**) Western blot analysis and statistical analysis of PKM2, iNOS, and Arg1 protein levels in LPS-stimulated peritoneal macrophages cultured with MSC-Exo or PKM2 siRNA. (**C**) Representative images of western blots and statistical analysis for the TSC2-mTOR-AKT signaling pathway in LPS-stimulated peritoneal macrophages treated with MSC-Exo or PKM2 siRNA. (**D**) Co-immunoprecipitation assays demonstrating the association of CD74 and PKM2 in peritoneal macrophages. (per group *n* = 3, Full-length blots/gels are presented in Supplementary Fig. 8)

In both mice and humans, the classical activation of M1 macrophages relies primarily on glycolytic metabolism [17]. Therefore, to further elucidate the mechanism by which CD74 modulates macrophage phenotypes, we focused on pyruvate kinase M (PKM), a key enzyme involved in glycolytic metabolism. To determine whether PKM1 or PKM2 is a target of CD74 in macrophages, the expression levels of PKM1 and PKM2 were measured by western blot in peritoneal macrophages treated with MSC-Exo or CD74 siRNA. The results revealed that PKM2 protein levels were significantly reduced, while PKM1 protein levels did not show significant changes in peritoneal macrophages treated with MSC-Exo or CD74 siRNA (Fig. 8A). Subsequently, the impact of PKM2 knockdown on macrophage phenotypes was investigated. Western blot analysis demonstrated that iNOS levels were significantly down-regulated and Arg1 levels were up-regulated after transfection with PKM2 siRNA in peritoneal macrophages (Fig. 8B). Additionally, the effects of PKM2 siRNA on TSC2-mTOR-AKT signaling pathways were investigated. In vitro experiments revealed that PKM2 siRNA exhibited similar regulatory effects on the TSC2-mTOR-AKT pathways as MSC-Exo in peritoneal macrophages (Fig. 8C) .These findings suggest that PKM2 is involved in CD74-mediated macrophage polarization by targeting the TSC2-mTOR-AKT signaling cascades.

To investigate how CD74 affects the expression of PKM2, experiments were carried out using peritoneal macrophages (Fig. 8D). The immunoprecipitation (Co-IP) experiments revealed a significant interaction between CD74 and PKM2. These findings strongly suggest that CD74 modulates the expression of PKM2 by interacting with PKM2.

## Discussion

This study confirms the protective effects of MSC-Exo against AngII or CaCl_2_-induced AAA formation and their ability to promote macrophage polarization towards the M2 phenotype. We further demonstrate that CD74 plays a crucial role in mediating MSC-Exo-induced macrophage polarization. Moreover, we uncover that CD74 regulates the downstream TSC2-mTOR-AKT pathway through its interaction with PKM2, thereby modulating macrophage polarization. These findings provide valuable insights into the therapeutic effects of MSC-Exo on AAA and highlight the critical involvement of CD74 in this process.

Macrophages have been shown to have both pathogenic and reparative roles in AAA, contributing to extracellular matrix remodeling, inflammation promotion and resolution, and tissue repair responses [3]. In our study, we discovered the critical involvement of macrophages in mediating the effects of MSC-Exo through a macrophage depletion model. In AAA formation, two distinct subgroups of macrophages, namely pro-inflammatory M1 macrophages and anti-inflammatory M2 macrophages, play significant roles [18]. Our study, using both an AAA mouse model and in vitro experiments, demonstrated that MSC-Exo effectively switched macrophages from an M1 to an M2 phenotype under inflammatory conditions. This phenotypic shift attenuated the inflammatory response and promoted tissue repair, ultimately inhibiting AAA development. Through protein mass spectrometry analysis and subsequent PCR validation, we identified CD74 as a potential effector involved in the immunomodulatory effects of MSC-Exo. Notably, there are currently no available studies investigating the relationship between CD74 and AAA, nor have the roles of CD74 in the immunomodulatory effects of MSC-Exo been explored. Our research provides novel insights by demonstrating, for the first time, that CD74 expression is negatively regulated by MSC-Exo in peritoneal macrophages, leading to the switching of macrophages towards the M2 phenotype.

Mechanically, bioinformatics analysis revealed that differentially expressed proteins in MSC-Exo-treated peritoneal macrophages were primarily enriched in metabolic pathways. Notably, PKM2, a key enzyme involved in glycolysis, has been implicated in the regulation of matrix metalloproteinase-9 (MMP9) activity and expression [19], which plays a critical role in AAA formation [20]. Therefore, we further investigated the association between CD74 and PKM2. Our data confirmed that inhibiting CD74 reduced PKM2 expression, and it was observed that CD74 could interact with PKM2. Additionally, inhibiting PKM2 resulted in a shift of macrophages from an M1 to an M2 phenotype, mimicking the beneficial effects seen with CD74 siRNA in vivo. Furthermore, our investigation revealed that CD74 and PKM2 impact the TSC2-mTOR-AKT pathway, with the potential to influence macrophage polarization mediated by MSC-Exo.

This study has several limitations that warrant further investigation. Firstly, additional studies are needed to explore the regulation of macrophage polarization by MSC-Exo through artificial manipulation of CD74 activation or inactivation at the cellular level. Secondly, the specific role of CD74 in macrophages in the pathogenesis of AAA is not fully understood and requires further investigation using cell type-specific knockouts and relevant physiological models. Lastly, our study focused on male mice, limiting the generalizability of the findings to female populations. Future studies should include both male and female subjects to assess any potential sex differences in the observed effects.

In summary, our study demonstrates that MSC-Exo protect against AAA formation by inducing a shift in macrophage polarization towards an anti-inflammatory phenotype. CD74 plays a crucial role in this process by modulating the PKM2-regulated TSC2-mTOR-AKT signaling pathway. These findings provide important insights into the therapeutic potential of MSC-Exo in AAA and highlight the significance of CD74 in regulating macrophage polarization. In future, more research using diverse models and conditions, along with corroboration through clinical studies or in vitro experiments using human samples, is needed to enhance the applicability of our findings.

## Conclusions

Our research indicate that MSC-Exo protect against abdominal aortic aneurysm formation through CD74 modulation of macrophage polarization in mice.

### Electronic supplementary material

Below is the link to the electronic supplementary material.

Supplementary Material 1

Supplementary Material 2

Supplementary Material 3

## Data Availability

We confirm that a protocol, including the research question, key design features, and analysis plan, was prepared before conducting the study and registered in Nanjing Drum Tower Hospital and all relevant data are included in the article and its supplementary materials or are available upon request.
