# Supplementary material for: Mesenchymal stromal cell-derived exosomes protect against abdominal aortic aneurysm formation through CD74 modulation of macrophage polarization in mice
Source: Stem Cell Res Ther. 2024 Aug 4;15:242. doi: 10.1186/s13287-024-03808-y (PMC11299418; doi:10.1186/s13287-024-03808-y)

**SUPPLEMENTAL METHODS**

**Animal experimental protocol**

1) AngII‑induced model: In this model, only male Apoe^-/-^ mice aged 8-10 weeks were used because female mice have a lower incidence of AngII-induced AAA. The mice were anesthetized using isoflurane (1.5-2%)and implanted subcutaneously with an osmotic pump (Alzet, MeDOl 2004) that released AngII at a rate of 1000 ng/kg•min for 4 weeks. During this period, the mice were fed a high-fat diet. As a control, Apoe^-/-^ mice were implanted with a normal saline osmotic pump and also fed a high-fat diet for 4 weeks. Monitoring the respiratory rate, body weight, and food intake of the mice is essential. If any signs of respiratory distress, significant weight loss, or lack of appetite are observed, the experiment will be terminated. After the modeling phase, the mice underwent abdominal ultrasound imaging to evaluate indicators of abdominal aortic aneurysm, including AAA incidence and maximum abdominal aortic diameter. An abdominal aortic aneurysm is defined as the abdominal aorta diameter exceeding 150% of the normal diameter. Two independent investigators, who were blinded to the experimental groups, analyzed the AAA incidence and maximum abdominal aortic diameter. Elastin van Gieson staining was performed to assess the degradation of elastic fibers in the aortas. The levels of iNOS and Arg1 were measured using western blot analysis, while the levels of iNOS and CD206 were detected through immunofluorescence staining in the aorta samples. These experiments were conducted to analyze the impact of the AngII-induced model on AAA development and associated molecular changes.

2) CaCl_2_-induced model: For the CaCl_2_-induced model, male C57BL/6 mice aged 8-10 weeks were anesthetized using isoflurane (1.5-2%). A midline incision was made in the abdomen to expose the renal artery. A gauze soaked with 0.5 mol/L CaCl2 was placed directly on the inferior segment of the abdominal aorta for 15 minutes. In the control group, a cotton gauze soaked with NaCl_2_ (0.9%) was used for a sham operation. All mice were fed a normal diet throughout the experiment. After 4 weeks, the mice were sacrificed, and AAA-related indicators were evaluated. In the macrophage depletion study, C57BL/6 mice were used. They received intravenous injections of 150 μL (5mg/mL) clodronate liposomes from Liposoma (The Netherlands). The injections were administered one day before and one day after inducing AAA. This method was utilized to deplete macrophages effectively in the experimental mice

For in vivo tracing of MSC-Exo, DiR-labeled MSC-Exo were injected into the tail veins of mice. In vivo imaging analysis was conducted at designated time points (2h, 24h, 48h, 72h, 5d, and 7d) after the injection to track the specific distribution of MSC-Exo in the mice.

To assess the maximum abdominal aortic diameter in mice, transabdominal ultrasonic detection was utilized. After 4 weeks of AAA model induction, mice were anesthetized with isoﬂurane gas for general anesthesia. Abdominal aortic imaging was performed using a 2-D mode transabdominal ultrasonic detector. The maximum abdominal aortic diameter was measured during the imaging process.

**Imaging of the Abdominal Aorta**

This study used the high-frequency small animal ultrasound system (Fujifilm VisualSonics) to detect the abdominal aortic model, with the probe used being MS400/MX400/UHF46x and a central frequency of 30MHz. Prepare the mouse for ultrasound by anesthetizing with O2 and isoflurane, ensuring proper positioning, preparing the skin, attaching electrodes for ECG readings, verifying heart rate, applying gel to the site, and positioning the probe for scanning.

Place the probe transversely just below the sternum and xiphoid process, with the reference marker facing the mouse’s right side. Next, locate the abdominal aorta adjacent to the inferior vena cava and/or portal vein, and confirm pulsatile flow using color Doppler. Adjust the ultrasound image crop for improved frame rate and change the focal depth to the posterior wall of the abdominal aorta. Then, move the probe caudally to visualize the branch points of the celiac and superior mesenteric arteries, using the right renal artery as a landmark. Finally, capture the region of interest showing maximum dilation in the abdominal aorta.

**Histological analysis**

After 4 weeks of modeling, the mice were euthanized, and their left ventricles were perfused with normal saline to remove blood. The aortic arch to iliac artery segments were dissected and fixed in 4% formalin. Abdominal aortic segments were then dehydrated, embedded in paraffin, and sliced into 5 μm thick sections.

For H.E. staining, the sections were stained with hematoxylin and eosin to examine the gross morphology of the vessels and measure the lumen diameter.

To assess the degradation of elastic fibers, paraffin sections were stained using a commercial kit (Solarbio, G1593).

For immunofluorescence staining, the deparaffinized tissue slides underwent antigen retrieval by boiling in citrate buffer at 100℃ for 1 hour. After blocking with 1% fetal bovine serum (FBS) for 1 hour at room temperature, the slides were incubated with primary antibodies overnight at 4℃. Following PBS washing, secondary antibodies were applied for 1 hour at room temperature. After another round of PBS washing, DAPI staining was performed for 10 minutes at room temperature. Finally, the slides were washed twice with PBS in the absence of light. Anti-fluorescence quencher was applied, and images were captured using a confocal microscope (Leica, TCSSP8) with a 200x objective.

**Cell isolation and culture**

Mouse bone marrow-derived MSCs were isolated from the tibia and femoral marrow compartments. The MSCs were cultured in MSC culture medium (Science Cell, 7501) supplemented with 5% FBS (Science Cell, 0025), 1% Penicillin/Streptomycin (Science Cell, 0503), and 1% mesenchymal stem cell growth supplement. MSCs between the 3rd and 6th generations were used for subsequent experiments.

Peritoneal macrophages were isolated from the peritoneal cavity of 8-week-old male BALB/c mice through intraperitoneal lavage using PBS. The cells were then filtered through a 70 μm mesh, lysed with ACK buffer (Invitrogen), and subsequently resuspended and plated in Dulbecco’s modified Eagle’s medium (DMEM) containing 10% FBS (Gibco) and 1% Penicillin/Streptomycin (HyClone). After incubating for 2 hours at 37°C and 5% CO2, the attached peritoneal macrophage cells were washed with PBS and cultured for further analysis.

Mouse macrophage RAW264.7 cells were cultured in DMEM supplemented with 10% FBS and 1% penicillin/streptomycin. The cultures were maintained at 37°C, 5% CO2, and 95% humidity.

**Labelling of exosomes**

To observe the internalization of MSC-Exo into macrophages, PKH67 dye was used to label the exosomes. The labeled MSC-Exo were washed with PBS and then subjected to centrifugation at 100,000×g at 4 °C for 1 hour. Subsequently, the PKH67-labeled MSC-Exo were co-cultured with RAW264.7 cells at a final concentration of 20 μg/ml. After 6 hours, the cells were washed with PBS and stained with DAPI (Ribobio). Finally, a confocal microscope (Leica, TCSSP8) was used to examine and capture images of the cells. The fluorescence intensity of the cells was analyzed using ImageJ software.

**SiRNA transfection**

siRNA transfection was performed on macrophages using Lipofectamine® 3000 (#L3000015, Thermo Fisher) following the manufacturer’s instructions. At 24 hours post-transfection, the efficiency of gene silencing was assessed by quantitative real-time PCR analysis(qRT-PCR).Subsequently, the impact of siRNA treatment on macrophage polarization was evaluated through various techniques such as qRT-PCR.The sequence are as TableS1.

**Cell** **immunofluorescence analysis**

The cells were fixed with paraformaldehyde for 10-15 minutes and washed 2-3 times with DPBS. Next, the cells were incubated with 0.1% Triton-100 for 5 minutes and washed again with DPBS. To prevent nonspecific binding, the cells were blocked with 5% BSA for 40 minutes at room temperature.For antibody staining, the cells were incubated overnight at 4 °C with the desired primary antibodies (diluted at 1:100-1:200) as listed in Supplemental Table 1. Following this, the cells were washed with PBS and incubated with secondary antibodies (diluted at 1:10,000) at room temperature in the dark for 60 minutes. DAPI staining was performed to label the nuclei, with a 10-minute incubation at room temperature.

Finally, the cells were washed with PBS and quickly imaged using a confocal microscope (Leica, TCSSP8). The fluorescence intensity of the cells was analyzed using ImageJ software.

**RNA isolation and quantitative real-time PCR (qRT-PCR)**

Total RNA was extracted from the cells using the TRIzol Reagent Invitrogen kit following the manufacturer’s instructions. From 1 μg of total RNA, cDNA was synthesized using the Transcriptor First Strand cDNA Synthesis Kit (Roche LifeScience).

For quantitative real-time PCR (qRT-PCR), the SYBR Green Reagents Kit (Roche LifeScience) was utilized. The mRNA levels were normalized to the housekeeping gene glyceraldehyde 3-phosphate dehydrogenase (GAPDH). Changes in expression were calculated using the ∆∆Ct method. Data were presented as the mean ± standard deviation (SD) obtained from at least three repeated experiments.The sequences of the primers used in qRT-PCR are as Table S2:

**Western blot analysis**

Proteins were isolated from cells or tissues using RIPA buffer supplemented with protease inhibitors (Sigma). The protein concentrations were determined using a BCA assay kit (ThermoFisher Scientific).For protein electrophoresis, the TGX FastCast acrylamide kit (BIO) was utilized. The proteins were separated by gel electrophoresis and then transferred onto a PVDF membrane (Millipore, Bedford, MA, America).The membranes were blocked with 5% skim milk for 2 hours and incubated overnight at 4°C with primary antibodies (diluted at 1:1000 or 1:500). GAPDH was used as the loading control.Afterwards, the membranes were washed three times for 10 minutes each with 0.1% TBS-T, followed by incubation with a horseradish peroxidase-conjugated secondary antibody (diluted at 1:10,000) for 2 hours. Protein bands were visualized using an ECL Prime detection system (GE).The antibodies used in the experiment are as Table S3:

**Flow cytometry analysis**

For tissue cytometry analysis, single-cell suspensions of tissues were obtained using the gentleMACS™ Dissociator from Miltenyi Biotec. The samples were then resuspended in staining buffer (R&D Systems) and incubated with F4/80-PerCP/Cy5.5 antibody from eBioscience for 30 minutes at 4°C.Flow cytometry analysis was performed using a FACS Aria flow cytometer from BD Bioscience. The acquired data were analyzed using FlowJo software from TreeStar (Ashland, OR). This software allows for the visualization and analysis of flow cytometry data.

**Enzyme-linked immunosorbent assay (ELISA)**

The supernatant of each group was extracted. The expressions of IL-1β and IL-6, IL-10 and TGF-β were detected with a specific ELISA kit (MultiSciences).

**Co-immunoprecipitation (****Co-IP) assay**

After pretreatment, the cells were washed twice with pre-cooled PBS. Then, 500 μl of ice-cold lysis buffer was added to the cells, which were kept on ice for 20 minutes. The lysates were centrifuged at 12,000×g for 10 minutes, and the supernatants were collected.

To perform immunoprecipitation, the supernatants were incubated with 2 μg of anti-CD74 antibody and anti-PKM2 antibody (from Abcam) or control IgG at 4 °C overnight with slow rotation. Following this, 25 μl of prewashed protein A/G beads were added to the mixture and incubated for an additional 2 hours.After three washes with pre-cooled PBS, the resulting lysate was used for western blot analysis to detect the specific proteins of interest.

**4D Label-free Proteomics analysis**

The cells were categorized into two experimental groups: LPS+PBS and LPS+MSC-exo. After 24 hours of exosome and PBS stimulation, the culture medium was discarded. The dish was inverted onto absorbent paper to remove excess medium, followed by the addition of pre-cooled PBS at 4°C. Gentle shaking of the dish for 1 minute facilitated thorough washing of the cells, after which the PBS was discarded. This washing process was repeated twice to eliminate any remaining culture medium. The petri dish was placed on ice, and 4°C pre-cooled PBS was added. Using a clean cell scraper, the cells on one side of the dish were quickly scraped, and then the dish was tilted on ice to allow the buffer to flow to one side. The lysate was pipetted into a pre-cooled centrifuge tube and subjected to centrifugation to remove the supernatant. Subsequently, the sample was frozen in liquid nitrogen for subsequent 4D Label-free Proteomics analysis performed by Shanghai GENE CHEM CO.LTD.

Table S1 The siRNA involved sequence of this research.

| Name | sense（5'-3'） | antisense（5'-3'） |
| --- | --- | --- |
| Jpt1 | GGGAGAAGGUGAUAUGCAUTT | AUGCAUAUCACCUUCUCCCTT |
| Ppp1r2 | GCGGCCUCAACGGCCUCGCTT | GCGAGGCCGUUGAGGCCGCTT |
| S100a13 | UGAACAUCAAUGAAUUUAATT | UUAAAUUCAUUGAUGUUCATT |
| Psme3ip1 | GAGGAGCAAUUCAAAUUCATT | UGAAUUUGAAUUGCUCCUCTT |
| Ccnh | GUCACAGUUACUGGAUAUATT | UAUAUCCAGUAACUGUGACTT |
| Dnase2 | CUUCUUCGCUCAGAAAUUATT | UAAUUUCUGAGCGAAGAAGTT |
| Rsad2 | GCGUGGAAGAAGCAAUAAATT | UUUAUUGCUUCUUCCACGCTT |
| Tgoln1 | GGCCACAGAAGAUGAUUCUTT | AGAAUCAUCUUCUGUGGCCTT |
| Clec4e | GGGAGCCCAACAAUAUAGUTT | ACUAUAUUGUUGGGCUCCCTT |
| Fxyd5 | GCGACUACUCGUGACAAUGTT | CAUUGUCACGAGUAGUCGCTT |
| Klra2 | GCUCCUUAAUGGAAGAAAUTT | AUUUCUUCCAUUAAGGAGCTT |
| CD74 | GGACAUGGAAGACCUAUCUTT | AGAUAGGUCUUCCAUGUCCTT |
| PKM2 | CAGAGACCAUCAAGAAUGUTT | ACAUUCUUGAUGGUCUCUGTT |

Table S2 The sequences of the primers used in qRT-PCR.

| Gene | Forward primer | Reverse primer |
| --- | --- | --- |
| Mus GAPDH | ATGATTCTACCCACGGCAAG | CTGGAAGATGGTGATGGGTT |
| Mus iNOS | TCACCTTCGAGGGCAGCCGA | TCCGTGGCAAAGCGAGCCAG |
| Mus Arg1 | CCAGATGTACCAGGATTCTC | AGCAGGTAGCTGAAGGTCTC |

Table S3 The western blot antibodies used in the experiment are as follows:

| Antibody | Source | Catalog number | Host species |
| --- | --- | --- | --- |
| CD63 | Abcam | Ab213090 | Mouse |
| CD9  TSG101  GAPDH  iNOS | Abcam  Abcam  proteintech proteintech | Ab92726  Ab125011  60004-1  18985-1-AP | Mouse  rabbit  mouse  rabbit |
| Arg1 | ABclonal | A4923 | rabbit |
| β-actin | ABclonal | AC038 | rabbit |
| CD74 | Abcam | Ab289885 | rabbit |
| TSC2 | ABclonal | A19540 | rabbit |
| Rheb  p-mTOR  t-mTOR  p-AKT  t-AKT  PKM2 | CST  ABclonal  ABclonal  Proteintech  Proteintech  Proteintech | #13879  AP0115  A2445  80455-1-RR  60203  60268 | rabbit  Rabbit  Rabbit  Rabbit  Mouse  Mouse |


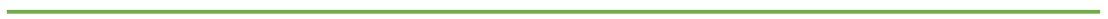


**Figure S1**

Figure S1 Systemic depletion of macrophages was achieved using clodronate (dichloromethylene diphosphonate, Cl2MDP) liposomes. Representative flow cytometry plots and statistical analysis were performed to assess the macrophage population in the spleen, blood, and aorta of mice treated with either PBS or clodronate liposomes.per group n=5. *****P<0.0001*

**Figure S2**

Figure S2 A.The Top 6 most significantly up-regulated proteins after MSC-exo stimulation of peritoneal macrophages(Jpt1, Ppp1r2, S100a13, Psme3ip1, Ccnh, and Dnase2). B. The mRNA level of iNOS and Arg1 in LPS-stimulated peritoneal macrophages cultured with MSC-Exo or MSC-Exo + siRNA treatment(Jpt1siRNA, Ppp1r2siRNA, S100a13 siRNA, Psme3ip1 siRNA, Ccnh siRNA, and Dnase2 siRNA)n=3. (**P* < 0.05, ***P* < 0.01, ****P* < 0.001, *****P* < 0.0001), ns=non-significance.

**Figure S3**

Figure S3 A.The Top 6 most significantly down-regulated proteins after MSC-exo stimulation of peritoneal macrophages(Rsad2, Tgln1, Clec4e, Fxyd5, CD74,and Klra2). B. The mRNA level of iNOS and Arg1 in LPS-stimulated peritoneal macrophages cultured with MSC-Exo or MSC-Exo + siRNA treatment(Rsad2siRNA, Tgln1 siRNA, Clec4e siRNA, Fxyd5 siRNA, CD74 siRNA,and Klra2 siRNA)n=3.(**P* < 0.05, ***P* < 0.01, ****P* < 0.001, *****P* < 0.0001), ns=non-significance.

**Figure S4**

MSC-Ex significantly reduced the expression of CD74 in mouse AAA tissues. A. Immunofluorescence staining was performed in the abdominal aortas of PBS- and MSC-Exo-treated AngII-induced AAA mice, employing CD74 (red), CD68 (green), and DAPI. (blue) (scale bars, 50 μm). B. Immunofluorescence staining was performed in the abdominal aortas of PBS- and MSC-Exo-treated CaCl_2_-induced AAA mice, employing CD74 (red), CD68 (green), and DAPI. (blue) (scale bars, 50 μm)

Full-length blots/gels are presented as follows:


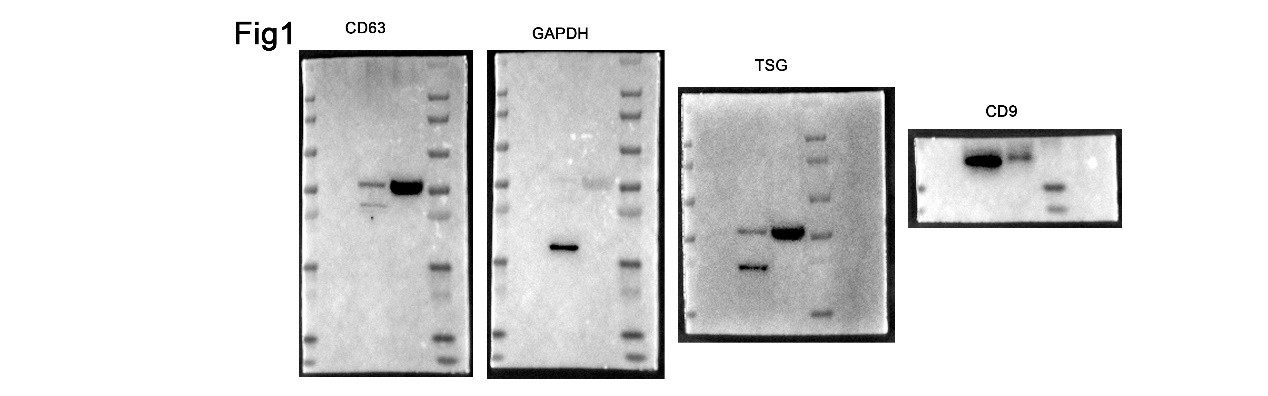


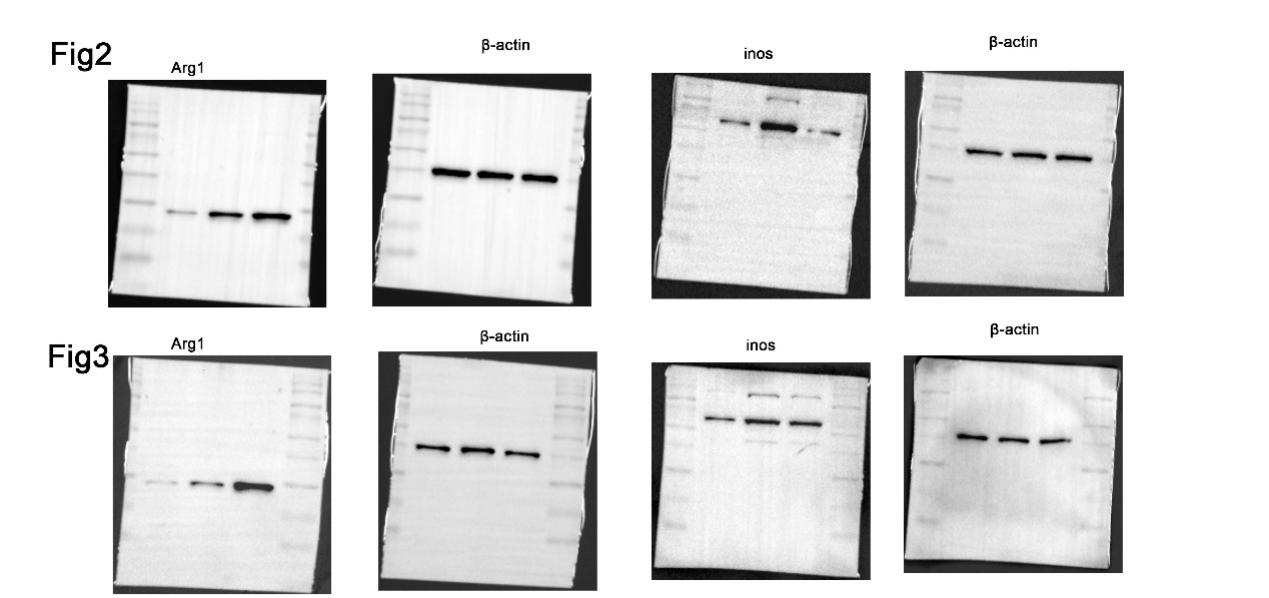


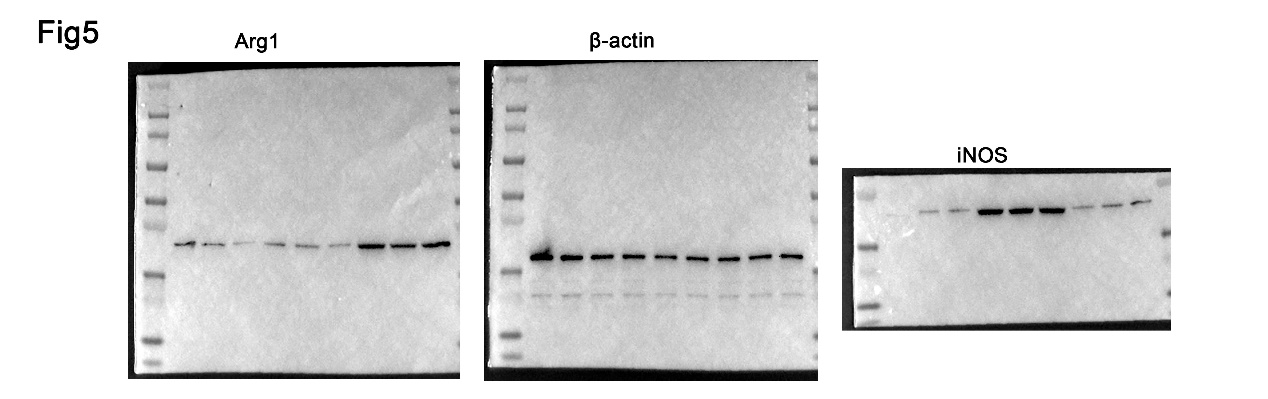


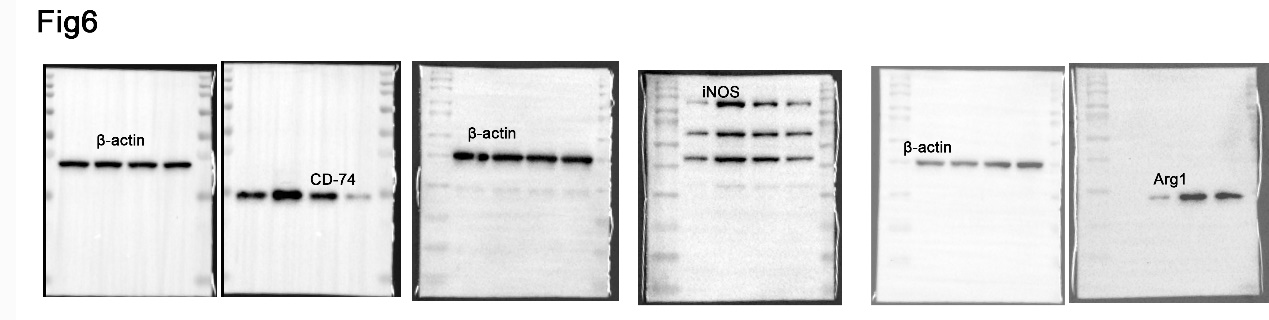


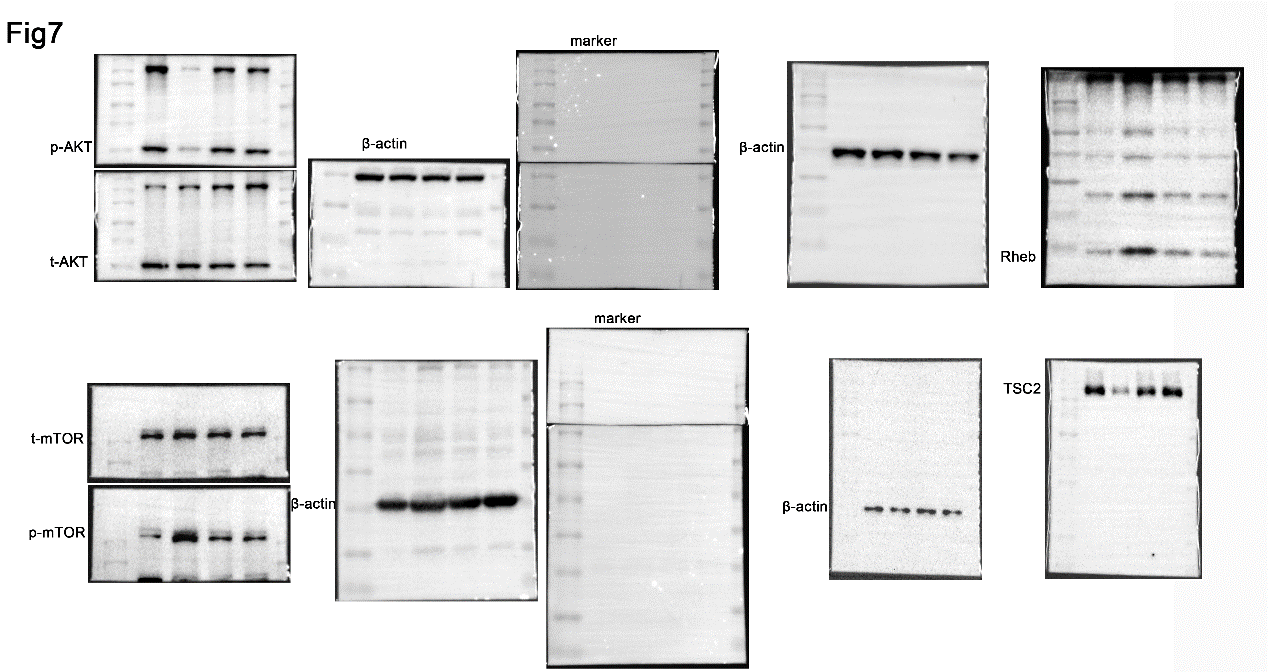


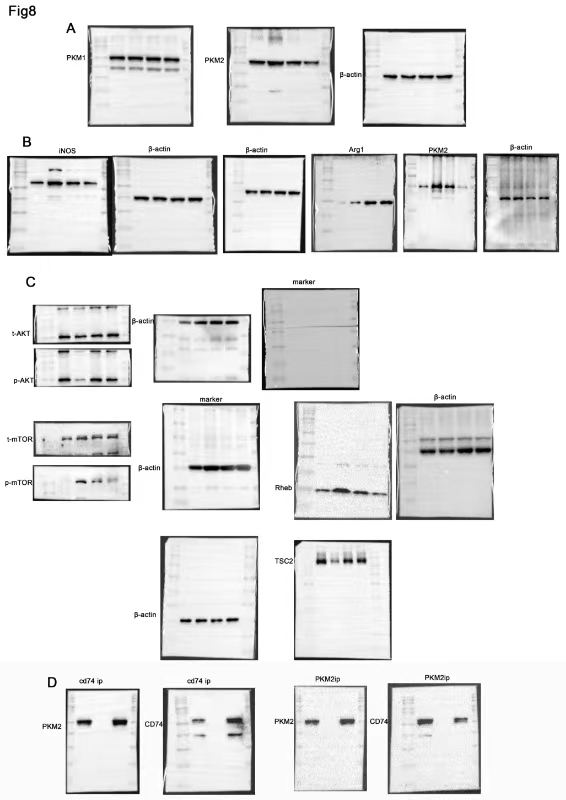

Supplement: Supplementary file 1 — Supplementary Material 1 [file 13287_2024_3808_MOESM1_ESM.docx]
