# Supplementary material for: Mesenchymal stromal cell-derived exosomes protect against abdominal aortic aneurysm formation through CD74 modulation of macrophage polarization in mice
Source: Stem Cell Res Ther. 2024 Aug 4;15:242. doi: 10.1186/s13287-024-03808-y (PMC11299418; doi:10.1186/s13287-024-03808-y)

**Figure S1** Systemic depletion of macrophages was achieved using clodronate (dichloromethylene diphosphonate, Cl2MDP) liposomes. Representative flow cytometry plots and statistical analysis were performed to assess the macrophage population in the spleen, blood, and aorta of mice treated with either PBS or clodronate liposomes.per group n=5. ****P<0.0001


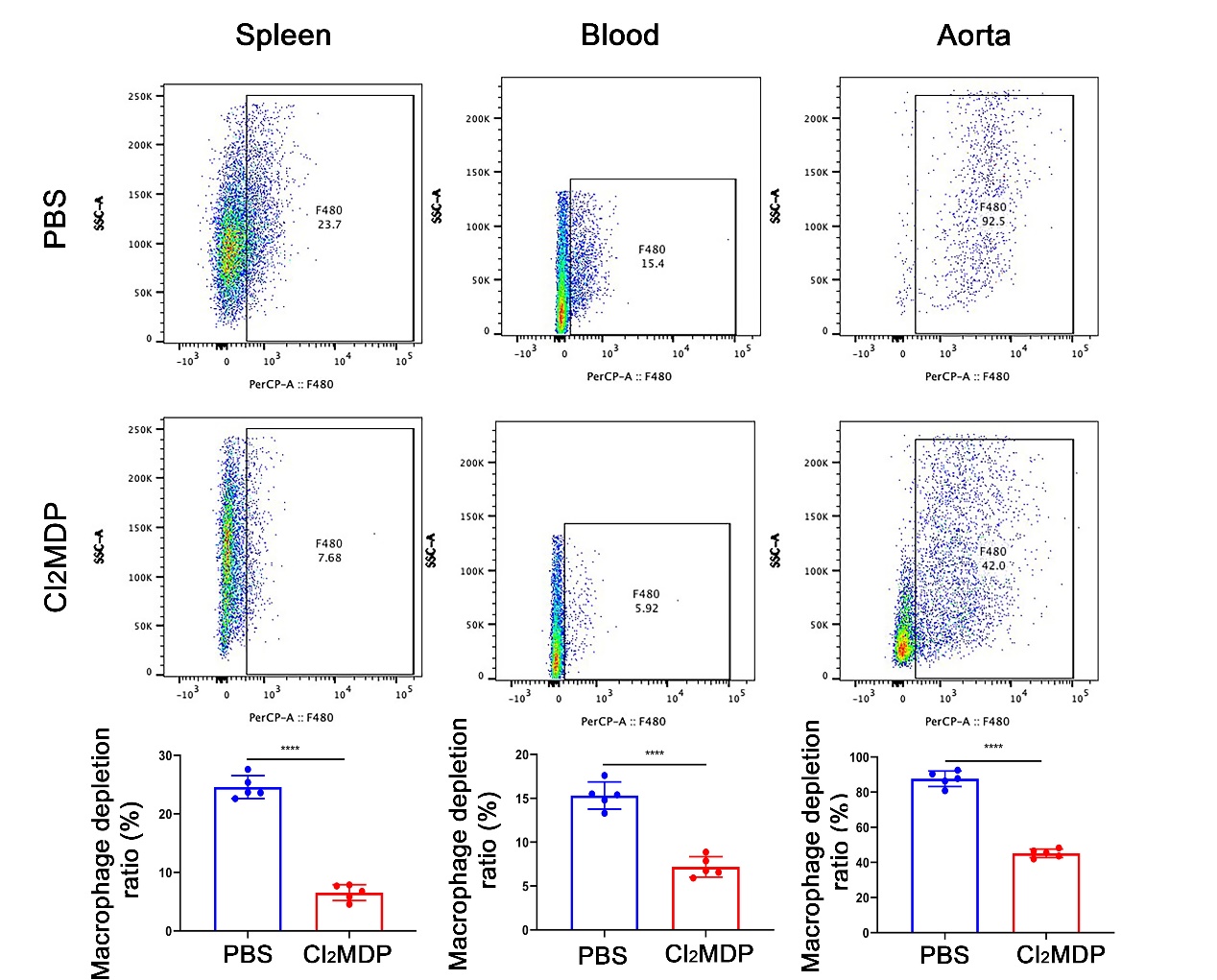


**Figure S2** A.The Top 6 most significantly up-regulated proteins after MSC-exo stimulation of peritoneal macrophages(Jpt1, Ppp1r2, S100a13, Psme3ip1, Ccnh, and Dnase2). B. The mRNA level of iNOS and Arg1 in LPS-stimulated peritoneal macrophages cultured with MSC-Exo or MSC-Exo + siRNA treatment(Jpt1siRNA, Ppp1r2siRNA, S100a13 siRNA, Psme3ip1 siRNA, Ccnh siRNA, and Dnase2 siRNA)n=3. (*P < 0.05, **P < 0.01, ***P < 0.001, ****P < 0.0001), ns=non-significance.


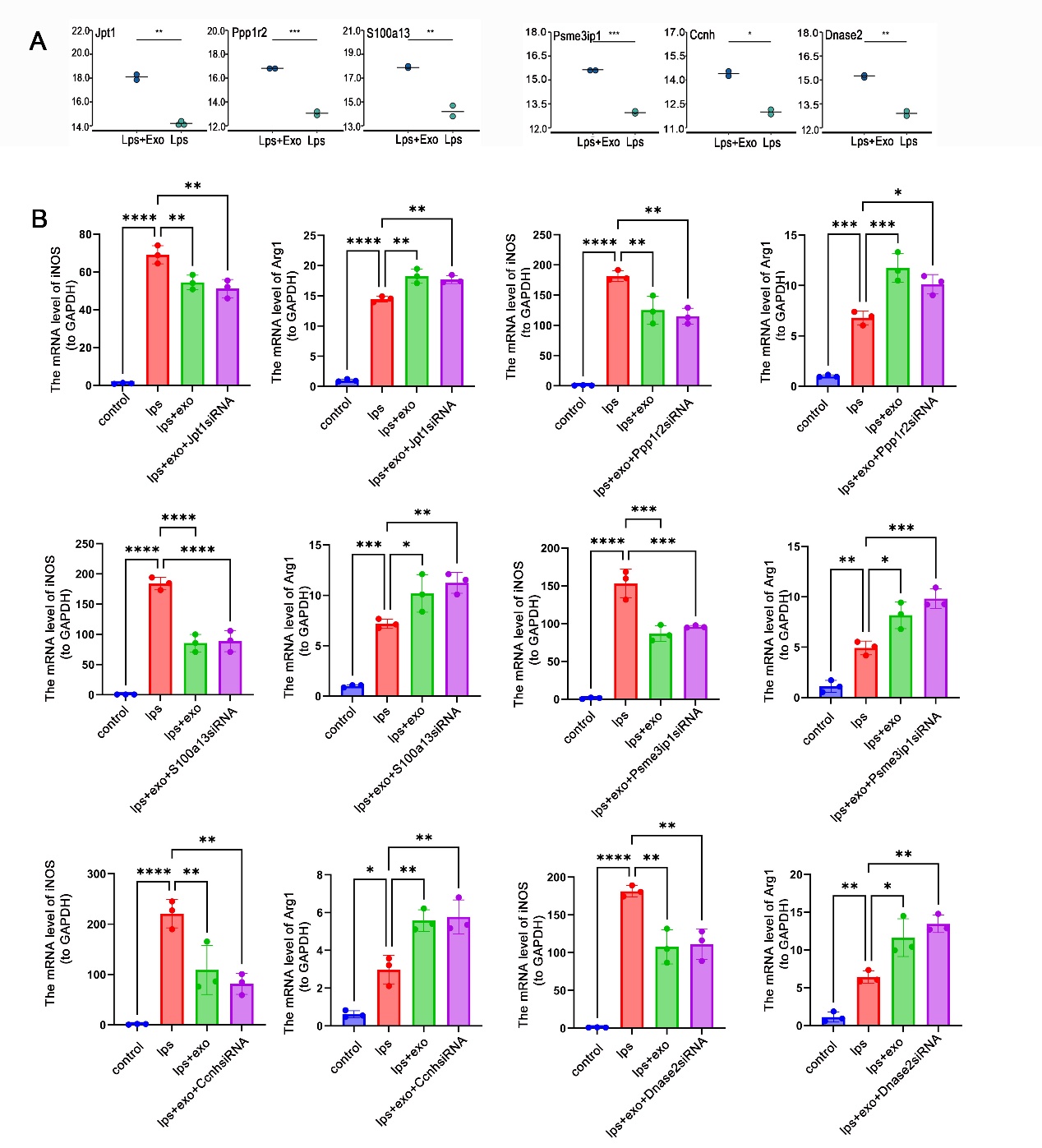


**Figure S3** A.The Top 6 most significantly down-regulated proteins after MSC-exo stimulation of peritoneal macrophages(Rsad2, Tgln1, Clec4e, Fxyd5, CD74,and Klra2). B. The mRNA level of iNOS and Arg1 in LPS-stimulated peritoneal macrophages cultured with MSC-Exo or MSC-Exo + siRNA treatment(Rsad2siRNA, Tgln1 siRNA, Clec4e siRNA, Fxyd5 siRNA, CD74 siRNA,and Klra2 siRNA)n=3.(*P < 0.05, **P < 0.01, ***P < 0.001, ****P < 0.0001), ns=non-significance.


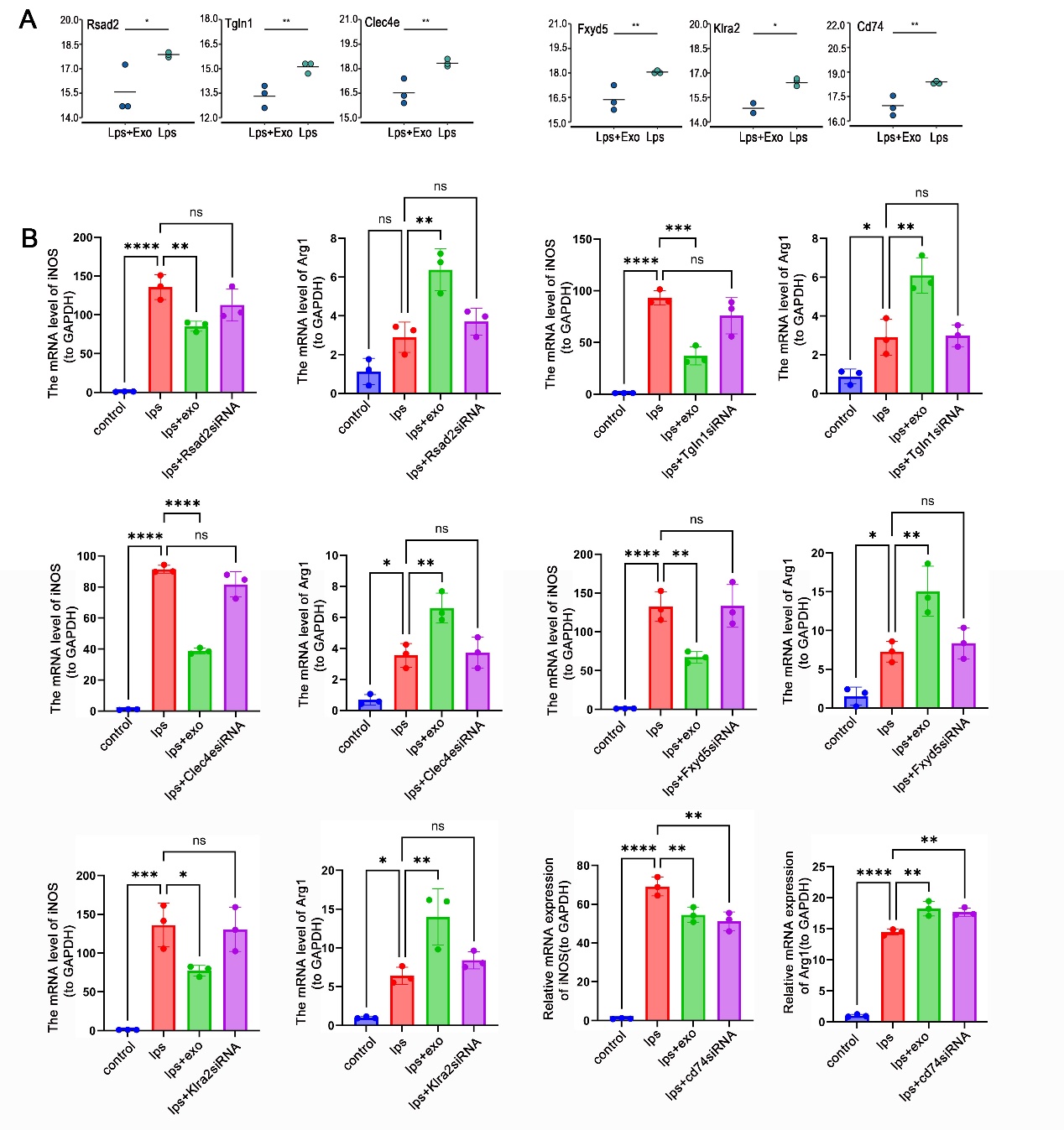


**Figure S4** MSC-Ex significantly reduced the expression of CD74 in mouse AAA tissues. A. Immunofluorescence staining was performed in the abdominal aortas of PBS- and MSC-Exo-treated AngII-induced AAA mice, employing CD74 (red), CD68 (green), and DAPI. (blue) (scale bars, 50 μm). B. Immunofluorescence staining was performed in the abdominal aortas of PBS- and MSC-Exo-treated CaCl2-induced AAA mice, employing CD74 (red), CD68 (green), and DAPI. (blue) (scale bars, 50 μm)


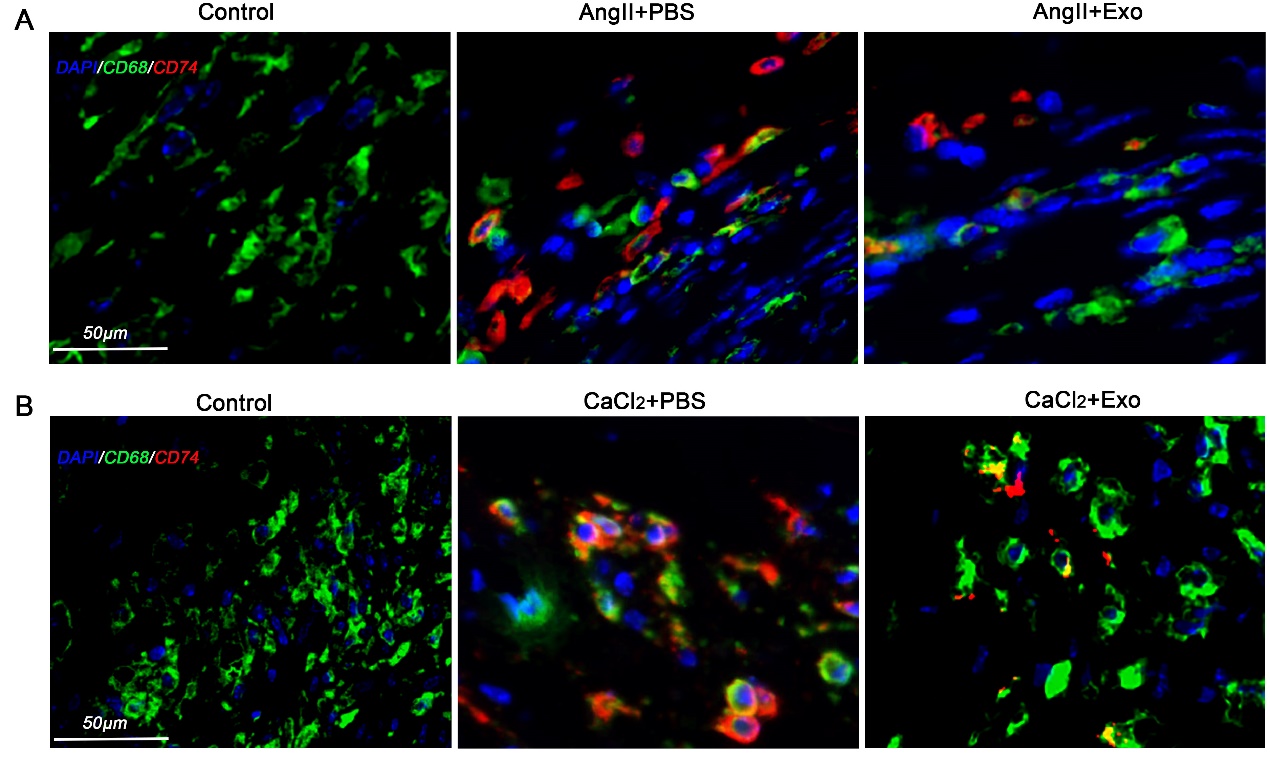

Supplement: Supplementary file 3 — Supplementary Material 3 [file 13287_2024_3808_MOESM3_ESM.docx]
